# Covalent Organic Frameworks for CO_2_ Capture: From Design to Application

**DOI:** 10.3390/nano16120777

**Published:** 2026-06-19

**Authors:** Hafezeh Nabipour, Sohrab Rohani

**Affiliations:** Department of Chemical and Biochemical Engineering, University of Western Ontario, London, ON N6A 5B9, Canada

**Keywords:** covalent organic frameworks, carbon capture, gas adsorption, porous materials

## Abstract

The increasing concentration of atmospheric CO_2_ has intensified the urgent need for efficient and sustainable carbon capture technologies. Covalent organic frameworks (COFs) have emerged as a highly promising class of porous crystalline materials for CO_2_ adsorption and separation owing to their structural tunability, high surface area, and precisely designable pore environments. This review summarizes recent advances in COF-based CO_2_ capture systems, covering pristine COFs, functionalized frameworks, composite materials, and membrane-based architectures. In pristine COFs, CO_2_ adsorption is mainly governed by micropore confinement and physisorption within well-defined channels, where surface area and pore size distribution play key roles. Functionalized COFs introduce additional active sites, including amine groups, heteroatoms, ionic functionalities, and alkali metal centers, which significantly enhance CO_2_ affinity through stronger electrostatic and acid–base interactions, often leading to mixed physisorption–chemisorption behavior. Composite COFs and mixed-matrix membranes further improve performance through synergistic effects, interfacial engineering, and enhanced mass transport. Despite these advantages, challenges remain in achieving an optimal balance between capacity, selectivity, and regenerability under realistic conditions such as humidity, low CO_2_ partial pressure, and multicomponent gas streams. Issues related to scalable synthesis, structural stability, and processability also limit practical applications. Overall, this review highlights key structure–property relationships and outlines future directions, including humid-stable COFs, direct air capture, computational design strategies, and advanced membrane technologies, for next-generation CO_2_ capture materials.

## 1. Introduction

The expansion of the global economy is closely linked to increased fossil fuel consumption, leading to significant emissions of greenhouse gases, particularly CO_2_. Global CO_2_ emissions have risen by more than 50% compared to pre-industrial levels, contributing to intensified global warming and an increased frequency of extreme weather events and natural disasters such as droughts and floods. The Paris Agreement aims to limit the global temperature rise to below 2 °C, with efforts to restrict it to 1.5 °C above pre-industrial levels. Despite net-zero commitments by many countries by 2050, including China, France, Germany, and Japan, global CO_2_ emissions are still projected to reach approximately 22 billion tonnes by 2050, corresponding to a potential warming of about 2.1 °C by 2100. These projections highlight the urgent need for effective emission reduction strategies [1]. This alarming trend has prompted extensive research efforts and industrial initiatives aimed at mitigating CO_2_ emissions [2]. Among the various mitigation strategies, carbon capture and storage (CCS) is considered one of the most direct and feasible approaches, as it enables substantial reductions in CO_2_ emissions while remaining compatible with existing fossil-fuel-based energy infrastructures.

CO_2_ capture technologies can be broadly classified into three main routes: post-combustion, pre-combustion, and oxy-fuel combustion. These processes remove CO_2_ from flue gas streams or low-nitrogen combustion systems, each presenting specific technical challenges and energy penalties that can reduce overall system efficiency. Post-combustion capture is limited by low CO_2_ partial pressures, pre-combustion operates at high pressures with variable CO_2_ concentrations, and oxy-fuel combustion requires flue gas recycling to avoid extremely high flame temperatures. From a mechanistic perspective, CO_2_ separation methods include absorption, adsorption, membrane separation, cryogenic distillation, and chemical looping. Although absorption and chemical looping are widely applied, they often demand high energy input and specialized equipment, while membrane and cryogenic methods face high cost and operational limitations [3].

In contrast, adsorption-based technologies offer several advantages, including low energy consumption, operational simplicity, and environmental compatibility [3]. Adsorption also allows the rational design of tailored materials, providing high selectivity and efficiency in CO_2_ separation while maintaining relatively low operational and maintenance costs. Consequently, considerable attention has been devoted to the development of optimal CO_2_ adsorbents [3,4]. An ideal adsorbent generally exhibits several essential characteristics: (i) a high specific surface area and well-developed porous structure; (ii) excellent chemical and thermal stability under diverse environmental conditions; and (iii) strong affinity toward target CO_2_ molecules in multicomponent gas mixtures [5]. Over the past decades, a broad spectrum of materials has been investigated for CO_2_ capture, including chemisorption-based systems (primarily amine solutions), metal oxides, biomaterials, boron nitride, MXenes, basic salts, layered double hydroxides, ionic liquids, and various porous materials such as activated carbon, zeolites, metal–organic frameworks (MOFs), and covalent organic frameworks (COFs) [1,6]. Natural zeolites are porous aluminosilicate minerals with reversible water adsorption–dehydration performance, high chemical and thermal stability, good mechanical strength, and moderate CO_2_ capture capacity and selectivity. These properties make them attractive candidates for CO_2_ adsorption. However, zeolites are easily saturated with water, which significantly reduces their CO_2_ uptake under humid conditions. Additionally, non-uniform pore size distribution limits their performance at both low and high pressures, restricting their large-scale industrial application [7].

Molecular sieves are synthetic hydrated aluminosilicates designed with molecular screening functions. They exhibit high adsorption capacity, strong selectivity, and excellent thermal resistance. Adsorption in molecular sieves primarily occurs via van der Waals forces, while their strong polarity and internal Coulombic fields enhance uptake of polar and unsaturated molecules. Despite these advantages, molecular sieves have very small pore sizes, usually less than 2 nm, and their rigid inorganic frameworks make it challenging to introduce specific functional modifications [7].

COFs are porous materials constructed from organic building blocks containing light elements such as carbon, boron, oxygen, hydrogen, and nitrogen. These materials can incorporate diverse structural motifs, including benzene rings [8], imine linkages [9], triazine units [10], and porphyrin-based frameworks [11]. COFs can be synthesized through various approaches, such as mechanochemical synthesis, ionothermal synthesis, solvothermal methods, and microwave-assisted techniques [12]. Owing to their high surface area, tunable pore architecture, and chemical robustness, COFs have attracted increasing scientific attention for a wide range of applications, including gas storage and separation [13], heterogeneous catalysis [14], energy storage [15], biotechnology [16], pollution mitigation [17], electrocatalytic reduction [18], and optoelectronic applications [19]. Benefiting from the abundant designability of organic monomers, the crystallinity and long-range order of covalent frameworks, and the diversity of covalent linkages, COFs exhibit significant advantages over conventional porous materials such as zeolites, molecular sieves, porous carbons, and MOFs. These advantages include low framework density, high surface area, tunable pore size and geometry, and versatile post-synthetic functionalization, which collectively make COFs highly promising candidates for CO_2_ capture applications [20]. In addition, the structural flexibility of COFs enables the incorporation of various functional groups while maintaining crystallinity, allowing continuous tuning of adsorption sites and interaction strength. Importantly, several COFs also demonstrate stable cyclic CO_2_ uptake performance and good resistance under humid conditions, highlighting their potential for practical gas separation processes [21].

Compared with MOFs, COFs generally exhibit superior chemical stability due to the absence of labile metal–ligand coordination bonds and the presence of robust covalent linkages. In particular, many COFs remain thermally stable above 300 °C and show high resistance toward a wide range of organic solvents, provided that their covalent backbone is preserved. Moreover, some COFs possess exceptionally high surface areas exceeding 2400 m^2^/g, which further contributes to their high adsorption capacity and efficiency for CO_2_ capture [20].

From a broader perspective, COFs offer a distinctive balance between the robust structural stability of zeolites and the high chemical tunability of MOFs. Zeolites are well known for their excellent thermal and chemical stability; however, their fixed pore systems and limited possibilities for structural modification often restrict their performance in selective CO_2_ capture, particularly under variable operating conditions. In contrast, MOFs provide exceptionally high surface areas and a wide range of structural diversity, yet their practical application is frequently constrained by moderate hydrothermal stability and susceptibility to degradation in humid or acidic environments. COFs, which are constructed entirely from light elements such as C, H, N, O, and B, bridge this gap by combining strong chemical stability with an inherently modular and designable architecture. This allows fine control over pore size, topology, and surface functionality, enabling systematic tuning of CO_2_ adsorption sites while maintaining structural integrity. In addition, because adsorption in most COFs is dominated by physisorption, they generally require lower regeneration energy compared with chemisorption-based systems, which facilitates easier CO_2_ desorption and more efficient cyclic operation. Nevertheless, their relatively low framework density can result in reduced volumetric uptake compared with many MOFs, and challenges remain in terms of pelletization, processability, and large-scale manufacturing. Accordingly, COFs occupy an intermediate yet strategically important position between highly stable zeolites and highly tunable MOFs, making them promising candidates for next-generation CO_2_ capture technologies [20,21,22]. While they integrate the stability of zeolites with much of the design flexibility of MOFs, limitations in volumetric performance and industrial scalability still need to be addressed.

Unlike many previous reviews that have primarily focused on COF synthesis, structural development, or adsorption performance in isolation, this work adopts a more integrated structure–property–performance perspective for CO_2_ capture. Special attention is given to how linkage chemistry, pore architecture, heteroatom incorporation, functionalization strategies, and membrane integration collectively govern adsorption capacity, selectivity, and regeneration behavior. Recent advances in pristine COFs, functionalized COFs, composite systems, and COF-based membranes are also critically compared in order to extract general design principles for future high-performance CO_2_ capture materials. Overall, this chapter aims to provide a comprehensive overview of COF design, synthesis, functionalization, and CO_2_ adsorption behavior while also highlighting current challenges and future research directions in this rapidly evolving field.

## 2. Fundamentals of COFs

### 2.1. Structure of COFs

The development of COFs marks a significant advance in the design of extended crystalline materials constructed entirely through covalent bonds. These frameworks are assembled from multifunctional organic monomers whose geometry, symmetry, and chemical functionality dictate the overall topology, dimensionality, and stability of the resulting networks. COFs are typically formed via reversible condensation reactions, which allow dynamic error correction during assembly and promote the formation of highly ordered crystalline structures rather than amorphous polymers. Early examples employed boronic acids and esters to generate boroxine and boronic ester linkages, while subsequent developments introduced more robust motifs, such as imines, hydrazones, azines, and β-keto-enamines, enhancing chemical and hydrolytic stability. By carefully selecting the building blocks and the type of linkage chemistry, it is possible to construct two- or three-dimensional frameworks with well-defined porosity, high crystallinity, and remarkable thermal and chemical resilience, making COFs versatile platforms for a wide range of practical applications [21,22,23,24].

### 2.2. Classification of COFs

**Figure 1:**
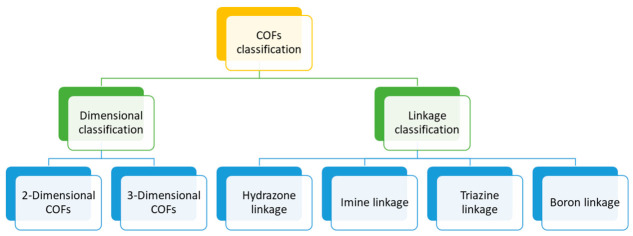
Schematic representation of the classification of COFs.

COFS can be categorized according to two main criteria: their dimensionality and the type of chemical linkage connecting the building blocks. Based on dimensionality, COFs are divided into two-dimensional (2D) and three-dimensional (3D) networks. Regarding the type of linkage, the frameworks are typically classified into boron-based, triazine-based, imine-based, and hydrazone-based COFs, as illustrated in Figure 1.

#### 2.2.1. Dimensional Classification of COFs

##### 2D COFs

**Figure 2:**
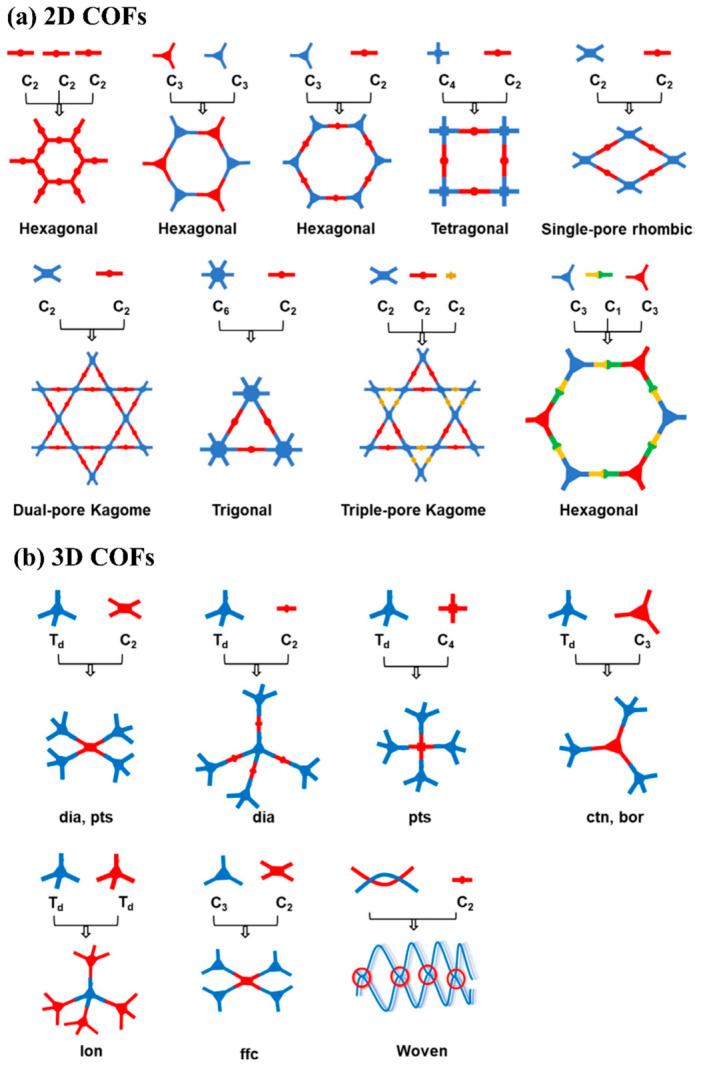
Representative (**a**) 2D and (**b**) 3D COF topologies generated from building units with different connectivities and symmetries. Arrows denote the assembly of the corresponding building blocks into the resulting network topologies. In the woven topology, red circles indicate strand-crossing (interlacing) points characteristic of the woven framework architecture. Reproduced from Ref. [28] with permission from MDPI, 2023.

2D COFs consist of stacked planar sheets forming extended layered networks. This arrangement facilitates the creation of ordered channels for π-electron delocalization, which can enhance charge transport along the stacked planes. These materials can achieve exceptionally high surface areas, reaching up to 3000 m^2^/g. As illustrated in Figure 2a, the specific topology of a 2D COF depends on the symmetry and connectivity of its building blocks: combining C3–C2 or C3–C3 units generates a hexagonal lattice, while C4–C2 and C4–C4 units produce tetragonal frameworks. Connections between C2–C2 units result in rhombic structures, and C6–C2 combinations form trigonal lattices, illustrating the diversity of 2D architectures [25,26,27].

3D COFs are fabricated using a tetrahedral arrangement of monomeric building blocks. 3D COFs represent a surface area of up to 5000 m^2^/g. These frameworks are constructed using tetrahedral (T_d_) or orthogonal monomeric units combined with multiple symmetric constituents, which facilitate backbone adjunction into 3D networks. Well-defined topologies like ctn, bor, pts, dia, and srs originate from configurations like T_d_–C_3_, T_d_–T_d_, T_d_–C_2_, and T_d_–C_4_ (Figure 2b). Among these, the dia arrangement is the most predominant one because of the great diversity of interoperable and adaptable organic linkers, which showcase the significance of progressive building blocks and compelling bonding techniques in 3D frameworks of COFs. If any of the COF monomers is three-dimensional, then the synthesized framework will also be 3D [25,26,27].

#### 2.2.2. Linkages in COFs

COFs are primarily defined by their connectivity, which originates from the linkage of organic building blocks through specific covalent bonds. A key feature of COFs is their crystallinity, which is largely governed by the reversible nature of bond formation during synthesis. In some cases, COFs are also constructed via irreversible covalent reactions. In systems based on reversible chemistry, the dynamic bond formation enables self-healing, structural optimization, and functional tuning, ultimately leading to highly crystalline frameworks [29,30,31]. Based on the type of covalent linkage formed, COFs can be classified into several categories, including boronate ester-based frameworks, imine-linked frameworks, hydrazone-linked frameworks, azine-linked frameworks, and other linkage types [29].

##### Boron Linkage

**Figure 3:**
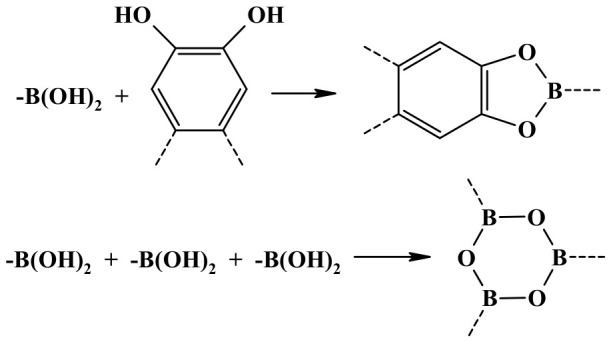
Formation of boroxine and boronate ester linkages.

COFs containing boron linkages are assembled primarily through boroxine ring formation or boronate ester condensation. As shown in Figure 3, in self-condensation, three boronic acid molecules lose three water molecules to form a planar B_3_O_3_ boroxine ring, while co-condensation involves boronic acids reacting with polyols to create boronate ester linkages between layers. Both reactions are dynamic covalent processes that generate water as a byproduct, enabling error correction and high crystallinity under solvothermal conditions with controlled water/alcohol presence. The electron-deficient boron centers act as Lewis acids, driving condensation but also rendering them susceptible to nucleophilic hydrolysis by water. Consequently, boron-based COFs exhibit excellent stability in organic solvents but typically undergo rapid hydrolytic degradation in aqueous environments. Recent strategies—including post-synthetic oligoamine modification, hydrophobic coatings, and stabilizing co-monomers—have significantly enhanced their water resistance while preserving porosity and crystallinity [32].

In 2005, Yaghi’s group introduced the first COF, COF-1 [(C_3_H_2_BO)_6_·(C_9_H_12_)_1_], by forming a boroxine-linked network through the self-condensation of 1,4-benzenediboronic acid. This initial breakthrough was followed by the development of COF-5 (C_9_H_4_BO_2_), which was obtained via a condensation reaction between 2,3,6,7,10,11-hexahydroxytriphenylene and 1,4-benzenediboronic acid. Both frameworks display exceptionally high porosity, with measured Brunauer–Emmett–Teller (BET) surface areas of approximately 711 and 1590 m^2^/g, respectively [26].

##### Hydrazone Linkage

**Figure 4:**
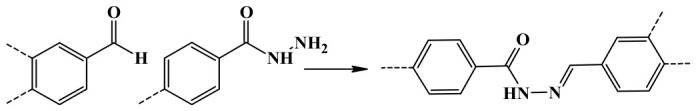
Hydrazone linkage formation.

**Figure 5:**
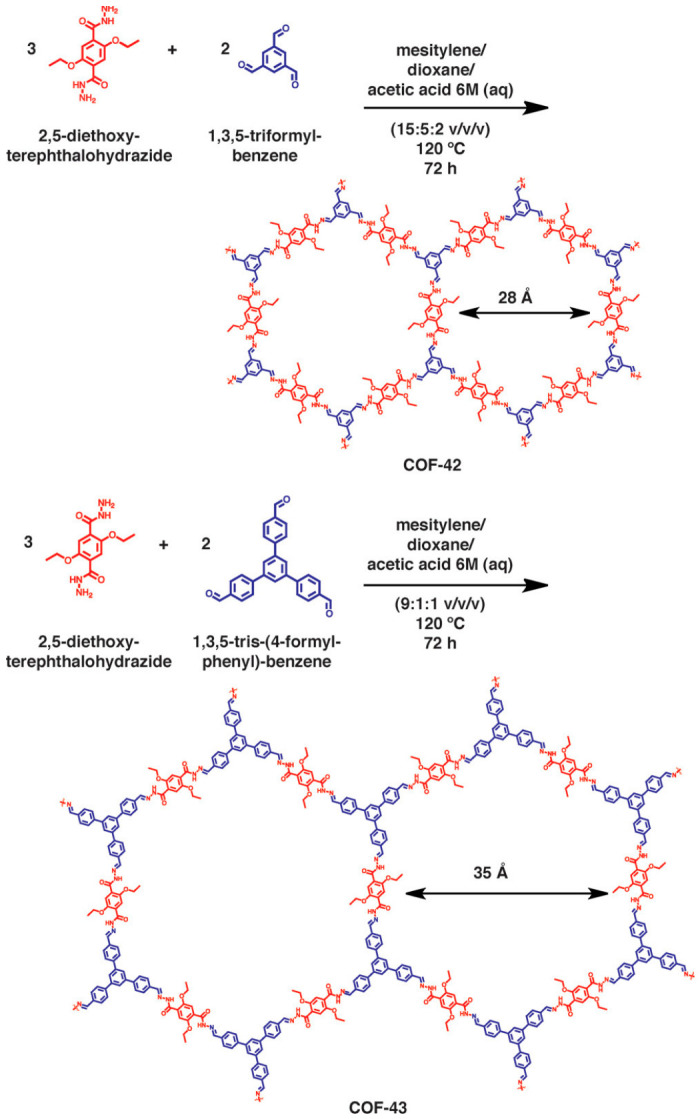
Illustrates the synthesis of COF-42 and COF-43 via condensation reactions between linear 2,5-diethoxyterephthalohydrazide linkers (red) and trigonal planar aldehyde nodes, namely 1,3,5-triformylbenzene or 1,3,5-tris(4-formylphenyl)benzene (blue), resulting in frameworks with distinct cavity sizes. Reproduced from Ref. [34] with permission from ACS, 2011.

Hydrazone-linked COFs are formed through a reversible condensation between aromatic aldehydes and hydrazide-based building units, generating acylhydrazone (C=N–NH) bonds with the release of water, as shown in Figure 4. This transformation is typically promoted by mild acid catalysts such as acetic acid, allowing dynamic bond exchange that supports structural error correction and improved crystallinity. Although the application of hydrazide precursors can be constrained by limited solubility and synthetic challenges—making the structural diversity largely dependent on the aldehyde component—the resulting hydrazone frameworks are generally more resistant to hydrolysis than imine analogues. Furthermore, extensive hydrogen-bonding interactions within the acylhydrazone linkage favor eclipsed layer stacking and contribute to the notable thermal and chemical stability of hydrazone-based COFs [33]. In 2011, Yaghi and co-workers reported the synthesis of two new hydrazine-linked two-dimensional COFs, COF-42 and COF-43, obtained through the condensation of 2,5-diethoxyterephthalohydrazide linkers with trigonal planar aldehyde nodes, namely 1,3,5-triformylbenzene and 1,3,5-tris(4-formylphenyl)benzene (Figure 5). The resulting frameworks exhibit high crystallinity, permanent porosity, and remarkable resistance to chemical and thermal degradation. This work expanded the structural diversity of hydrazine-derived two-dimensional COFs and presented a promising platform for adsorption-related applications [34].

##### Triazine Linkage

**Figure 6:**
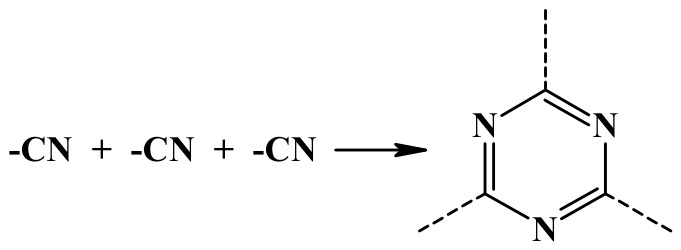
Triazine linkage formation.

Figure 6 depicts the chemical pathways involved in the formation of triazine linkages. Triazine-based COFs can be synthesized through the cyclotrimerization of aromatic nitrile groups, in which three –C≡N moieties react to form a thermodynamically stable triazine ring. This process is commonly conducted under ionothermal or acid-catalyzed conditions, enabling the construction of extended networks interconnected by strong C–N bonds. An alternative synthetic route involves the condensation reaction between aldehydes and amidines, leading to triazine ring formation through stepwise C–N bond generation and elimination of small molecules. Although triazine-linked COFs generally display lower crystallinity compared to boron-linked analogues due to the irreversible nature of the bond-forming reactions, they exhibit markedly enhanced chemical and thermal stability. Furthermore, the high nitrogen content within the triazine units provides abundant coordination sites, making these frameworks particularly attractive as catalyst supports and for applications requiring strong metal–framework interactions [33]. Triazine-based covalent organic frameworks (CTFs) were initially reported by Thomas and colleagues in 2008 using an ionothermal synthesis strategy. In this approach, nitrile-containing precursors undergo cyclotrimerization in molten ZnCl_2_ at approximately 400 °C, leading to the formation of the CTF-1. This framework exhibits a hexagonal architecture with one-dimensional pore channels and a BET surface area of about 1000 m^2^/g [35]. CTF materials have also been prepared through a condensation pathway involving aldehydes and amidine dihydrochlorides. This process proceeds via initial Schiff base formation, followed by a Michael addition, ultimately yielding porous organic frameworks. Using this strategy, CTF-HUST-1 was synthesized from 1,4-phthalaldehyde and terephthalamidine dihydrochloride, exhibiting a BET surface area of 663 m^2^/g and a pore volume of 0.32 cm^3^/g [36].

##### Azine Linkage

**Figure 7:**
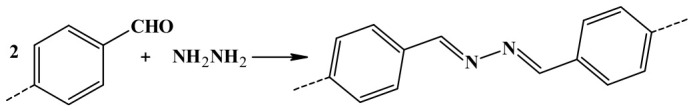
Azine linkage formation.

The formation of azine- or hydrazine-linked COFs is based on a thermodynamically controlled condensation reaction between hydrazine units and aldehyde groups, producing C=N–N=C linkages with the elimination of water, as illustrated in Figure 7. The reversibility of this reaction enables dynamic bond exchange, which promotes high crystallinity in the framework. This chemistry also contributes to the development of porous networks with enhanced structural order and stability [37]. In a recent study, a novel three-dimensional COF featuring azine linkages (3D-HNU5) was synthesized via the condensation of hydrazine with tetrakis(4-formylphenyl)methane in the ionic liquid [Bmim][Tf_2_N] (1-butyl-3-methylimidazolium bis(trifluoromethylsulfonyl)imide) at room temperature. The resulting material exhibited a BET surface area of 864 m^2^/g and a total pore volume of 0.89 cm^3^/g and was evaluated for CO_2_ capture applications.

##### Imine Linkage

**Figure 8:**
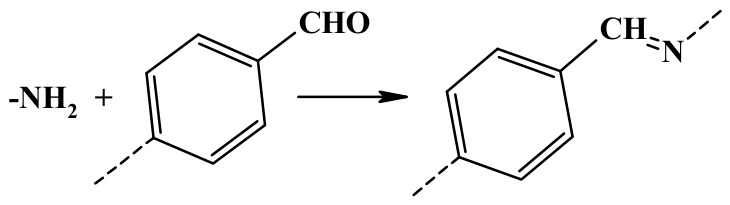
Imide linkage formation.

Imine-linked COFs are formed via the condensation of amino and aldehyde groups, commonly derived from p-phenylenediamine and benzaldehyde derivatives. The reaction initially produces amorphous polyimine precipitates, which gradually rearrange into crystalline COF aggregates through reversible imine condensation that helps correct structural defects (Figure 8). Additives such as acetic acid and water play a crucial role: acetic acid regulates precipitation, while water facilitates imine exchange, improving crystallinity. Although imine-linked COFs are generally more stable than boron-based COFs in water and organic solvents, their crystallinity often remains lower. Strategies like introducing interlayer hydrogen bonding or complementary electronic interactions have proven effective in enhancing both crystallinity and porosity [38]. For example, the porous and crystalline COF-HNU2 for CO_2_ uptake, with a surface area of 4313 m^2^/g, was synthesized via the condensation of 2-hydroxy-benzene-1,4-dialdehyde and 1,3,5-tris(4-aminophenyl)benzene [39]. In 2009, Yaghi and co-workers reported the synthesis of a 3D imine-based COF, COF-300, through the condensation of terephthaldehyde with tetra-(4-anilyl)methane. This imine-based COF featured a diamond-like 3D network, with an average pore size of 7.8 Å and a BET surface area of 1360 m^2^/g. Thermogravimetric analysis (TGA) under nitrogen showed that COF-300 remained stable up to 490 °C, while PXRD (powder X-ray diffraction) measurements confirmed its high crystallinity [40].

##### Other Linkage-Based COFs

Beyond the linkage chemistries described above, the development of additional functionalized COFs remains essential to satisfy the demands of diverse applications. Consequently, a wide variety of linkage-engineered COFs have been constructed through precise control of framework topology, pore characteristics, and functional properties. For instance, EL-Mahdy et al. reported the preparation of three [3 + 3] β-keto-enamine-linked COFs, namely TFP-TPA-COF, TFP-Car-COF, and TFP-TPP-COF, obtained via solvothermal polycondensation between tris(aminophenyl)-containing precursors bearing amino (TPA), carbazole (Car), or pyridine (TPP) functionalities and 1,3,5-triformylphloroglucinol (TFP-3OHCHO). These materials exhibited high crystallinity together with BET surface areas reaching 686 m^2^/g, and their structural morphology and building-block planarity were found to significantly influence CO_2_ adsorption behaviour [41]. In a separate study, imide-linked COFs were synthesized from mellitic trianhydride (MTA) combined with either 1,3,5-tris(4-aminophenyl)benzene (TAPB) or 1,3,5-tris(4-aminophenyl)amine (TAPA), yielding MTI-COF-1 and MTI-COF-2, respectively. These frameworks displayed BET surface areas of 339 and 397 m^2^/g, accompanied by measurable CO_2_ uptake capacities [42].

Collectively, these examples highlight the rapid expansion of COF linkage chemistry in recent years. In general, boronate-ester-linked COFs provide excellent crystallinity but suffer from hydrolytic instability. Imine-linked COFs exhibit improved resistance toward aqueous environments and maintain stability in many organic media, although their crystallinity is typically reduced. Triazine-based COFs, while often less crystalline than boronate analogues, usually demonstrate superior chemical robustness and thermal endurance. Therefore, selecting an appropriate linkage motif is fundamentally governed by the intended application of the COF material.

## 3. Properties of COFs

Porosity is a fundamental factor governing CO_2_ adsorption in COFs, as their ordered channels, large internal surface areas, and tunable pore environments provide abundant binding sites and efficient diffusion pathways for gas molecules. COFs display diverse pore geometries—such as tetragonal, hexagonal, trigonal, kagome, and rhombic structures—covering micro- to mesoporous regimes that enable precise control over adsorption behavior. In particular, micropores and ultramicropores with widths below ~1 nm enhance host–guest interactions and promote strong CO_2_ uptake at low pressure, while appropriate tuning of pore size and volume improves selectivity over competing gases like N_2_ and H_2_. Nevertheless, excessively large pores or very high porosity can reduce the density of effective adsorption sites and weaken interactions with CO_2_, indicating the need for an optimal structural balance. Experimental examples including azine-linked ACOF-1, COF-JLU-2, TpPa-COF, and HEX-COF-1—with pore sizes around 0.9–1.3 nm—demonstrate notable CO_2_ adsorption even without pore-wall functionalization. Incorporating nitrogen-rich or amine functionalities into the pore surfaces further strengthens CO_2_ binding, highlighting the synergistic interplay between controlled porosity and surface chemistry in achieving high adsorption capacity and selectivity [3,20,43,44].

### 3.1. CO_2_ Adsorption Mechanisms in COFs

#### 3.1.1. Physisorption vs. Chemisorption

**Figure 9:**
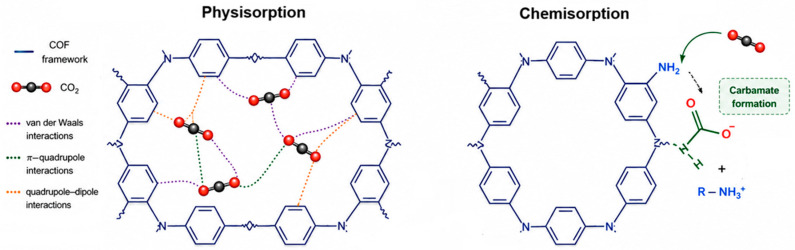
Comparison of physisorption and chemisorption mechanisms for CO_2_ capture in COFs.

CO_2_ adsorption in COFs is primarily governed by physisorption and chemisorption, depending on the chemical nature of the pore environment and functional groups (Figure 9). In physisorption, CO_2_ interacts weakly with the pore surface through van der Waals forces, π–π interactions, and electrostatic quadrupole interactions, leading to reversible binding and low regeneration energy requirements. In contrast, chemisorption occurs when CO_2_ reacts with functional groups such as amines to form carbamate or bicarbonate species, resulting in stronger binding and enhanced selectivity but higher regeneration energy demand. The balance between these mechanisms is crucial: physisorption ensures fast kinetics and easy regeneration, while chemisorption improves selectivity under dilute CO_2_ conditions [45,46,47].

#### 3.1.2. Role of Pore Confinement

Pore confinement is a key factor governing CO_2_ adsorption in COFs, because their ordered and tunable pore structures strongly influence host–guest interactions. In microporous and ultramicroporous COFs, the close proximity between the pore walls and adsorbate molecules enhances adsorption potential, especially at low pressures, where confinement effects become more important. This is particularly relevant for CO_2_ capture because narrow pores can increase the overlap between the gas molecule and the pore surface, thereby strengthening adsorption under dilute conditions [43].

Pore size also affects molecular diffusion and selectivity. CO_2_ diffuses more readily into appropriately sized confined pores than larger or less strongly interacting gases, and pore-size tuning can therefore improve CO_2_/N_2_ separation performance. At the same time, excessively small pores may hinder mass transport and reduce accessibility to internal adsorption sites, while overly large pores can weaken confinement-driven interactions and lower uptake. Therefore, an optimal balance between pore size, pore connectivity, and framework accessibility is essential for maximizing both CO_2_ capacity and selectivity [48,49,50].

#### 3.1.3. Functional Group Effects

Surface functionalization is one of the most effective strategies for enhancing CO_2_ adsorption in COFs, because it allows precise tuning of the pore environment and strengthens host–guest interactions at the molecular level. The incorporation of polar or electron-rich groups such as –NH_2_, –OH, –COOH, –CN, and –NO_2_ can alter the local electronic structure of the framework and increase CO_2_ affinity through stronger dipole–quadrupole interactions and, in some cases, hydrogen bonding contributions. Amine-functionalized COFs are particularly important because they can interact selectively with CO_2_ and, depending on the chemical environment, may exhibit either reversible physisorption or stronger chemisorption. Post-synthetic modification and linker engineering are especially valuable since they can introduce functional groups without sacrificing crystallinity or porosity. Overall, functional-group engineering remains a central design principle for improving CO_2_ uptake and selectivity in COFs, especially under low-pressure or dilute-gas conditions [51,52,53,54].

#### 3.1.4. Thermodynamics of CO_2_ Adsorption

Thermodynamic properties play a central role in determining the efficiency and energy demand of CO_2_ capture in COFs. In cyclic adsorption processes, the total regeneration energy includes the heat of CO_2_ desorption, the sensible heat required to raise the adsorbent temperature, and, under humid conditions, the latent heat associated with water removal. Among these contributions, heat capacity is particularly important because lower values reduce the sensible heat penalty during regeneration; however, experimental heat-capacity data for COFs remain limited, leading to uncertainties in process simulation and design.

Another key parameter is the isosteric heat of adsorption (ΔH_ads_), which reflects the strength of CO_2_–framework interactions and directly influences both adsorption capacity and regeneration energy. For physisorption-dominated systems, ΔH_ads_ typically lies in the range of −25 to −35, whereas in stronger chemisorption interactions, it exceeds −50 kJ/mol. Therefore, an intermediate interaction strength is generally preferred to balance high CO_2_ uptake with feasible regeneration energy requirements. Structural features such as pore size distribution and functional-group incorporation can further tune adsorption thermodynamics by modifying the local electronic environment of the pore walls, enabling rational optimization of CO_2_ capture performance [50].

### 3.2. COFs for CO_2_ Adsorption

COFs are crystalline porous materials constructed from lightweight organic building blocks linked through strong covalent bonds. Since their introduction in 2005, they have attracted considerable attention due to their high surface area, tunable porosity, low density, and notable chemical stability. COFs are typically synthesized via reversible condensation reactions, which enable error correction during framework formation and promote the development of highly ordered crystalline structures. Post-synthetic activation is generally required to remove residual solvents and fully activate the porous network.

The CO_2_ adsorption performance of COFs is primarily governed by the chemistry of the building blocks, pore architecture, and linkage type, all of which can be systematically tuned to optimize gas uptake and selectivity. In particular, nitrogen-rich COFs and amine-functionalized frameworks exhibit enhanced CO_2_ affinity due to stronger interactions with the quadrupolar nature of CO_2_. Furthermore, the development of functionalized COFs, composite systems, and COF-based membranes has significantly expanded their applicability for efficient CO_2_ capture and separation. Typically, COFs exhibit CO_2_ uptake capacities in the range of approximately 2–6 mmol/g at 273 K and 1 bar, depending on their structural and chemical characteristics.

The crystalline order and porosity of COFs play a decisive role in determining their CO_2_ adsorption performance. PXRD analysis not only confirms the formation of highly ordered frameworks but also provides indirect evidence of continuous and well-defined pore channels, which are essential for efficient gas diffusion and accessibility of adsorption sites. A loss of crystallinity is often associated with framework collapse or partial amorphization, leading to blocked diffusion pathways and reduced CO_2_ uptake and selectivity. In parallel, BET-derived surface area and pore volume provide quantitative insight into the accessible internal surface; however, pore size distribution—particularly the presence of ultramicropores (<0.7 nm)—is more directly correlated with CO_2_ adsorption strength due to enhanced confinement effects and overlapping adsorption potentials. Therefore, the combined interpretation of PXRD and BET data enables a more rigorous structure–property relationship, linking long-range order and pore architecture with adsorption capacity, thermodynamics, and separation performance in COF materials.

#### 3.2.1. Pristine COFs

Pristine COFs provide a high degree of molecular-level designability, allowing precise control over pore architecture through the choice of organic building blocks with well-defined geometry and functionality. Through rational monomer design, it is possible to construct highly ordered porous networks with tunable pore sizes and diverse topologies. Although pristine COFs do not contain open metal sites for direct CO_2_ coordination, their adsorption behavior is largely influenced by heteroatom-containing functional groups embedded within the framework. In particular, heteroatoms such as nitrogen and sulfur enhance CO_2_ affinity through dipole–quadrupole interactions and localized electrostatic effects, which ultimately improve adsorption capacity and selectivity even in metal-free systems [47].

Song et al. reported a 3D imine-linked COF (JUC-610) constructed from tetrahedral and rectangular building blocks via a [4 + 4] Schiff-base condensation between tetra[(2-fluoro-4-aminophenyl)phenyl]methane and 1,2,4,5-tetrakis(4-formylphenyl)benzene. The framework exhibits high crystallinity with a twofold pts topology, as confirmed by PXRD analysis. The resulting material shows a high specific surface area of 2072 m^2^/g and uniform microporosity (~1.5 nm), both of which are favorable for gas adsorption. JUC-610 delivers CO_2_ uptake capacities of 55.97 cm^3^/g (2.50 mmol/g) at 273 K and 35.7 cm^3^/g (1.46 mmol/g) at 298 K. This behavior is attributed to the combined effect of micropore confinement and moderate host–guest interactions, reflected by a Q_st_ value of ~21.9 kJ/mol, enabling efficient yet reversible CO_2_ adsorption [55]. The adsorption process is mainly governed by the high surface area and accessible pore structure rather than specific chemical binding sites. The rigid 3D imine-linked network also contributes to structural stability and crystallinity, supporting efficient gas diffusion. Overall, this example shows that well-defined pore architecture alone can deliver considerable CO_2_ capture performance even without strong specific binding functionalities.

In 2020, Hu et al. reported pyrazine-cored imine-linked COFs (COF-H1 and COF-H2) synthesized via Schiff-base condensation between a nitrogen-rich tetratopic pyrazine-based amine and dihydroxyterephthalaldehyde linkers, yielding crystalline 2D microporous frameworks with quadrangular-pore structures. The incorporation of pyrazine nitrogen atoms and hydroxyl functional groups introduces abundant polar adsorption sites within the pore environment, which enhances CO_2_ affinity. COF-H1 exhibits a BET surface area of 510 m^2^/g, while COF-H2 shows a higher value of 625 m^2^/g; both frameworks possess uniform micropores of approximately 1.31 nm (COF-H1) and 1.18 nm (COF-H2). Regarding CO_2_ adsorption, COF-H1 shows a capacity of 56.8 mg/g (1.29 mmol/g) at 273 K and 30.3 mg/g (0.69 mmol/g) at 298 K, whereas COF-H2 exhibits slightly higher uptake values of 66.2 mg/g (1.5 mmol/g) at 273 K and 32.4 mg/g (0.74 mmol/g) at 298 K. The Q_st_ values are 32.38 kJ/mol for COF-H1 and 26.83 kJ/mol for COF-H2, indicating moderately strong interactions between CO_2_ molecules and the framework. The adsorption mechanism is mainly governed by dipole–quadrupole interactions between CO_2_ and nitrogen sites in the pyrazine and imine linkages, together with hydrogen-bonding interactions involving hydroxyl groups. Interestingly, despite the higher surface area of COF-H2, COF-H1 shows a stronger interaction energy, which is attributed to more effective intramolecular hydrogen bonding that improves the accessibility of nitrogen active sites for CO_2_ binding [56]. This system highlights the role of nitrogen-rich heterocycles and hydroxyl-functionalized pore environments in tuning CO_2_ capture performance in imine-linked COFs.

Das et al. [57] synthesized a nitrogen-rich covalent imine network (CIN) via Schiff-base condensation of melamine and 1,4-piperazinedicarboxaldehyde, generating an amorphous microporous framework containing abundant triazine and piperazine nitrogen sites. Despite its non-crystalline nature, the material exhibits permanent microporosity with a BET surface area of 722 m^2^/g and excellent thermal and chemical stability, remaining stable up to 623 K under both acidic and alkaline conditions. The high density of Lewis-basic nitrogen atoms significantly enhances the affinity toward CO_2_ molecules through quadrupole–dipole and Lewis acid–base interactions. As a result, the CIN achieves CO_2_ uptakes of 3.32 and 2.50 mmol/g at 273 and 298 K, respectively, along with exceptionally high CO_2_/N_2_ selectivities of 211 and 100 (IAST: Ideal Adsorbed Solution Theory) at the corresponding temperatures. The material also shows a high isosteric heat of adsorption for CO_2_ (Q_st_ = 42.4 kJ/mol), indicating strong host–guest interactions. The authors attribute the excellent adsorption performance to the synergistic effect of nitrogen-rich triazine and piperazine units combined with micropore confinement, which preferentially strengthens interactions with CO_2_ over N_2_ and CH_4_. These results demonstrate that introducing multiple Lewis-basic functionalities into porous organic frameworks is an effective strategy for enhancing CO_2_ capture and separation performance, even in amorphous porous networks [57].

Liu et al. [58] reported two new isoreticular 2D COFs with hcb topology, namely TP-TFPB-COF and TP-NFPB-COF, synthesized via [3 + 3] imine condensation between 2,4,6-trihydroxybenzene-1,3,5-tricarbaldehyde (Tp) and two C3-symmetric fluorinated amines: 5′-(4-aminophenyl)-2′,4′,6′-trifluoro-[1,1′:3′,1″-terphenyl]-4,4″-diamine (TFPB) and 5′-(4-amino-3,5-difluorophenyl)-2′,3,3″,4′,5,5″,6′-heptafluoro-[1,1′:3′,1″-terphenyl]-4,4″-diamine (NFPB). Both COFs show high crystallinity and permanent porosity with fluorinated pore environments, as confirmed by PXRD and gas sorption analysis. TP-TFPB-COF and TP-NFPB-COF exhibit maximum nitrogen adsorption capacities of 492 and 727 cm^3^/g, respectively, corresponding to BET surface areas of 871 and 1089 m^2^/g. Nonlocal density functional theory (NLDFT) pore size analysis further reveals narrow pore distributions centered at 1.42 nm and 1.48 nm, respectively. With increasing fluorine content in the framework, TP-NFPB-COF shows a change in CO_2_ adsorption behavior compared with TP-TFPB-COF, where CO_2_ uptake decreases from 47.9 (1.96 mmol/g) to 39.8 cm^3^/g (1.62 mmol/g) at 298 K. This trend suggests that fluorination tunes adsorption affinity by modifying pore polarity and host–guest interactions. At the same time, Q_st_ decreases from 23.4 to 12.9 kJ/mol, indicating weaker but more selective interactions in the highly fluorinated framework. Although the uptake is lower, TP-NFPB-COF shows improved separation performance, with IAST selectivity increasing from 2.04 to 2.86, which is attributed to the formation of a more polar and electronically heterogeneous pore surface. Grand canonical Monte Carlo (GCMC) simulations indicate that fluorine atoms act as preferential adsorption sites within the channels, facilitating directional C–H···F interactions and molecular discrimination. Density functional theory (DFT) calculations further confirm weak physisorption, with adsorption energies in the range of −0.107 to −0.127 eV, governed mainly by van der Waals and electrostatic interactions. Overall, these two COFs demonstrate that fluorine engineering can effectively regulate adsorption energetics, host–guest interactions, and separation selectivity while maintaining structural integrity and crystallinity [58].

**Figure 10:**
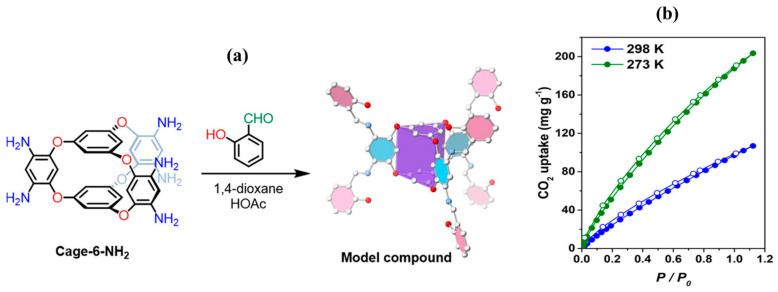
(**a**) Preparation of the model compound and its single-crystal structural analysis. In the single-crystal structure, carbon atoms are shown in gray, nitrogen in blue, and oxygen in red, while hydrogen atoms are omitted for clarity. (**b**) CO_2_ adsorption isotherms of 3D-CageCOF-1. Reproduced from Ref. [59] with permission from ACS, 2020.

Zhu et al. [59] reported the first 3D cage-based covalent organic framework, 3D-CageCOF-1, constructed via a dynamic [3 + 3] imine condensation between a trigonal prismatic organic cage node (Cage-6-NH_2_) and a linear linker (2,5-dihydroxyterephthalaldehyde, DHTPA) (Figure 10a). The incorporation of a hexafunctional cage unit enables true 3D connectivity, leading to an acs-type framework with a 2-fold interpenetrated structure, as confirmed by PXRD analysis and structural modeling. This work extends COF topology beyond conventional 2D layered systems. 3D-CageCOF-1 exhibits permanent microporosity with a BET surface area of 1040 m^2^/g and a pore volume of 0.50 cm^3^/g. The framework contains two types of well-defined micropores with diameters of approximately 5.6 Å and 8.8 Å, consistent with theoretical models. These confined channels are decorated with heteroatoms (N and O), creating a polar adsorption environment that is favorable for gas adsorption. A key feature of 3D-CageCOF-1 is its structural flexibility, showing reversible transformation between small-pore and large-pore phases upon guest adsorption/desorption, particularly with dimethylformamide (DMF). This breathing behavior arises from cooperative motion of the cage nodes, hinge-like flexibility of the organic backbone, and rotation of imine linkages, enabling adaptive pore regulation. The material shows strong CO_2_ adsorption capacity of 204 mg/g (4.46 mmol/g) at 273 K and 107 mg/g (2.43 mmol/g) at 298 K, attributed to the combination of narrow micropores and polar pore surfaces (Figure 10b). The adsorption process is mainly governed by physisorption through electrostatic interactions and hydrogen bonding within confined channels, which are further enhanced in the contracted (small-pore) state. No significant loss in performance is observed over repeated cycles, indicating good structural stability. 3D-CageCOF-1 highlights how organic cage-based nodes can generate new 3D COF topologies with hierarchical microporosity, structural dynamics, and enhanced adsorption performance, offering a promising strategy for next-generation porous materials [59].

Taken together, these representative studies suggest that no single structural parameter governs CO_2_ adsorption in pristine COFs. Instead, the adsorption behavior results from the combined influence of pore confinement, heteroatom chemistry, and pore surface polarity. Ultramicropores provide strong physical confinement at low pressures, while nitrogen-containing functional groups enhance adsorption enthalpy through dipole–quadrupole and electrostatic interactions. In contrast, excessive fluorination or weakly interacting pore environments can reduce adsorption efficiency by disrupting optimal host–guest interactions.

Overall, CO_2_ adsorption in pristine COFs follows a clear structure–property relationship in which micropore size (particularly ultramicroporosity), framework polarity, and heteroatom distribution work together. Among these factors, nitrogen incorporation consistently improves adsorption strength via dipole–quadrupole interactions, whereas pore confinement mainly determines capacity at low pressures. For this reason, the design of pristine COFs should focus on balancing pore geometry and chemical functionality, rather than simply maximizing surface area, in order to achieve both high uptake and favorable adsorption energetics.

Although pristine COFs show substantial CO_2_ uptake through physisorption and electrostatic interactions, their adsorption affinity and selectivity under dilute or humid conditions are still limited compared with chemisorption-based systems. Their moderate CO_2_–framework interaction strengths (Q_st_ ≈ 20–35 kJ/mol) are often not sufficient for highly selective capture at low partial pressures. To address these limitations, functionalization of COF frameworks has emerged as an effective strategy to introduce stronger and more specific CO_2_-binding sites, thereby improving adsorption affinity, selectivity, and low-pressure capture performance. The following section highlights representative functionalization strategies and their influence on CO_2_ adsorption behavior in COFs.

#### 3.2.2. Functionalized COFs

Functionalization of COFs is an effective strategy to address the limitations of pristine frameworks for CO_2_ capture. Introducing functional groups such as polar moieties and Lewis basic sites onto the pore walls enhances CO_2_ adsorption through stronger electrostatic and acid–base interactions. In particular, nitrogen- and oxygen-containing functionalities increase the density of active sites and improve CO_2_ affinity without significantly compromising the intrinsic porosity of the framework. These modifications also lead to improved adsorption performance and selectivity, making functionalized COFs more promising candidates for carbon capture applications compared to their pristine counterparts [47,60].

Wang et al. [61] prepared alkyl amine-functionalized triphenylamine-based COFs (TPA-COFs) as an efficient platform for CO_2_ capture and CO_2_/N_2_ separation through systematic pore wall engineering. In this system, a π-conjugated triphenylamine-based COF was post-synthetically modified with alkyl amine chains of different lengths (–(CH_2_)x–NH_2_, x = 1–4), allowing fine tuning of pore polarity and confinement without significantly disturbing the intrinsic crystallinity or high surface area of the framework (≈1800 m^2^/g). Structural analysis shows that increasing alkyl chain length gradually reduces pore size from ~1.5 nm to ~1.0 nm and decreases overall porosity while at the same time strengthening the electrostatic environment within the pores. Gas adsorption results reveal a clear dependence of CO_2_ uptake on alkyl chain length, following the order TPA-4C-NH_2_ > TPA-3C-NH_2_ > TPA-2C-NH_2_ > TPA-COF > TPA-1C-NH_2_. The optimal material, TPA-4C-NH_2_, shows the highest CO_2_ adsorption capacity of 5.26 mmol/g at 273 K and 3.41 mmol/g at 298 K (1 bar), along with a 121% increase in CO_2_/N_2_ selectivity compared to the pristine framework. This enhancement arises from a combination of stronger Coulombic interactions between CO_2_ molecules and amine-functionalized pore walls, stronger confinement in narrowed micropores, and increased framework polarity induced by longer alkyl chains. In addition, Q_st_ increases with alkyl chain length, confirming stronger host–guest interactions and more favorable CO_2_ binding at low coverage. In contrast, N_2_ adsorption is progressively suppressed due to its lower quadrupole moment and weaker interaction with the functionalized pore environment, which ultimately leads to improved selectivity. Importantly, the crystalline structure of the COF is fully preserved after functionalization, confirming the robustness of the framework. Overall, this study demonstrates that precise control over alkyl amine chain length is an effective strategy to simultaneously enhance CO_2_ uptake and separation performance in COF materials through combined pore confinement and electrostatic interaction effects [61].

From a broader perspective, these functionalization strategies highlight a key structure–property principle: while pristine COFs mainly rely on pore confinement, functionalized COFs introduce additional driving forces such as chemisorption, framework polarization, and site-specific electrostatic interactions. However, these effects do not act independently; instead, the overall adsorption performance results from a balance between increasing active site density and maintaining accessible porosity. This trade-off defines an optimal functionalization window for maximizing CO_2_ uptake and selectivity.

**Figure 11:**
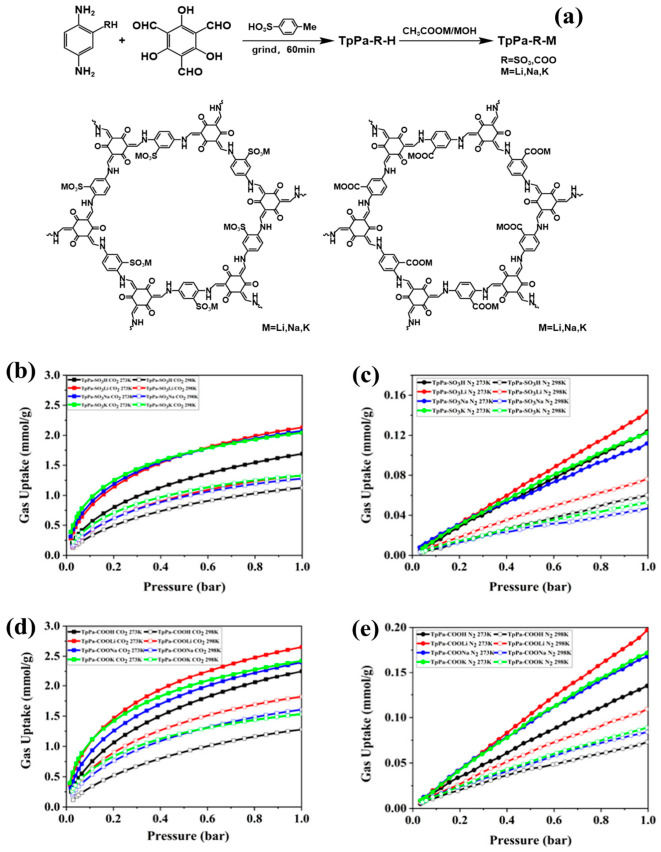
(**a**) Schematic illustration of the synthesis of TpPa-SO_3_H, TpPa-COOH, TpPa-COOR, and TpPa-SO_3_R (R = Li, Na, K). Adsorption isotherms of CO_2_ and N_2_ for functionalized TpPa-COFs and their alkali-metal derivatives at 273 and 298 K: (**b**) CO_2_ adsorption on TpPa-SO_3_H, TpPa-SO_3_Li, TpPa-SO_3_Na and TpPa-SO_3_K; (**c**) N_2_ adsorption on TpPa-SO_3_H, TpPa-SO_3_Li, TpPa-SO_3_Na and TpPa-SO_3_K; (**d**) CO_2_ adsorption on TpPa-COOH, TpPa-COOLi, TpPa-COONa and TpPa-COOK; and (**e**) N_2_ adsorption on TpPa-COOH, TpPa-COOLi, TpPa-COONa and TpPa-COOK at pressures up to 1 bar. Reproduced from Ref. [62] with permission from ACS, 2025.

Alkali metal-functionalized COFs are generally prepared through a two-step strategy involving initial framework construction followed by post-synthetic ion exchange. In the case of TpPa-COOH and TpPa-SO_3_H, β-ketoenamine-linked COFs are first formed via mechanochemical or condensation-based routes, which ensures high chemical stability of the resulting frameworks (Figure 11a). Subsequent ion exchange with alkali metal salts (Li^+^, Na^+^, K^+^) introduces metal cations into the –COO^−^ and –SO_3_^−^ functional groups while maintaining the original crystallinity and topology, as confirmed by PXRD analysis. Textural characterization shows that TpPa-COOR (R = H, Li, Na, K) exhibits BET surface areas of 216, 96, 101, and 65 m^2^/g, respectively, while NLDFT analysis reveals corresponding pore sizes of 1.48, 1.37, 1.26, and 1.18 nm (Figure 11b). Similarly, TpPa-SO_3_R (R = H, Li, Na, K) shows BET surface areas of 91, 65, 67, and 65 m^2^/g, with pore sizes of 1.37, 1.35, 1.34, and 1.27 nm, respectively (Figure 11c). These trends indicate a gradual decrease in pore size upon alkali metal incorporation, which can be attributed to partial pore occupation by metal ions. To evaluate CO_2_ adsorption capacity and CO_2_/N_2_ separation performance, single-component adsorption isotherms were measured at 273 and 298 K over a pressure range of 0–1 bar. TpPa-COOR (R = Li, Na, K) shows higher CO_2_ uptake than TpPa-SO_3_R, mainly due to its larger surface area and more accessible pore structure. At 273 K and 1 bar, CO_2_ uptake values for TpPa-COOR are 2.65 (Li), 2.40 (Na), and 2.42 mmol/g (K), compared with 2.25 mmol/g for TpPa-COOH. At 298 K, the corresponding values are 1.82, 1.60, and 1.53 mmol/g, respectively, higher than TpPa-COOH (1.28 mmol/g) (Figure 11d). A similar trend is observed for TpPa-SO_3_R, where CO_2_ capacities at 273 K are 2.13 (Li), 2.08 (Na), and 2.05 mmol/g, compared with 1.69 mmol/g for TpPa-SO_3_H. At 298 K, the values increase to 1.33, 1.28, and 1.33 mmol/g, respectively, versus 1.13 mmol/g for the parent material (Figure 11e). The enhanced CO_2_ uptake is mainly attributed to alkali metal-induced framework polarization and stronger electrostatic interactions with CO_2_ molecules. Among the metal ions, Li^+^ shows the most pronounced effect due to its higher charge density, which enables stronger charge polarization and more favorable interaction sites. In contrast, Na^+^ and K^+^ provide slightly weaker enhancement because of their lower charge density and larger ionic radius, which reduces interaction strength. Overall, TpPa-SO_3_R materials show lower CO_2_ uptake compared with TpPa-COOR due to their smaller surface areas and more constrained pore environments. However, alkali metal incorporation still improves their performance relative to TpPa-SO_3_H through increased framework polarization. In both series, CO_2_ uptake generally follows the order Li ≥ Na ≈ K. Additionally, the stronger electron-withdrawing nature of –SO_3_^−^ groups contributes to higher intrinsic polarity, partially compensating for the reduced surface area [62].

In another study, an amine-functionalized COF (TaTp-COF) was synthesized via Schiff-base condensation followed by spontaneous enol–to–ketoamine tautomerization, which introduces a high density of uniformly distributed secondary amine (–NH–) groups within the framework [63]. XRD analysis confirms the high crystallinity of TaTp-COF, which adopts an AA-stacked layered structure, with good agreement between simulated and experimental patterns, indicating well-ordered π–π stacking between 2D layers. This structural transformation is accompanied by a decrease in BET surface area (from 758 to 114 m^2^/g) and an increase in pore size (from 0.77 to 1.15 nm). Despite the reduced surface area, TaTp-COF shows a significantly enhanced CO_2_ adsorption capacity, reaching up to 5.0 mmol/g at 0 °C and 1 bar, outperforming the imine-linked TaTb-COF (3.1 mmol/g). This clearly indicates that CO_2_ uptake is not solely controlled by textural properties but is strongly influenced by chemical functionality. The incorporation of –NH groups increases the electron density at nitrogen sites and enhances the basicity of the framework, leading to stronger host–guest interactions with CO_2_. CO_2_-TPD and spectroscopic analyses suggest a transition from physisorption in TaTb-COF to chemisorption in TaTp-COF, associated with the formation of carbamic acid (–NHCOOH) species. This is further supported by in situ FTIR and solid-state nuclear magnetic resonance (NMR) results, which show consumption of –NH groups and the appearance of characteristic C=O signals after CO_2_ adsorption. Accordingly, the isosteric heat of adsorption is higher for TaTp-COF, confirming stronger binding affinity [63].

**Figure 12:**
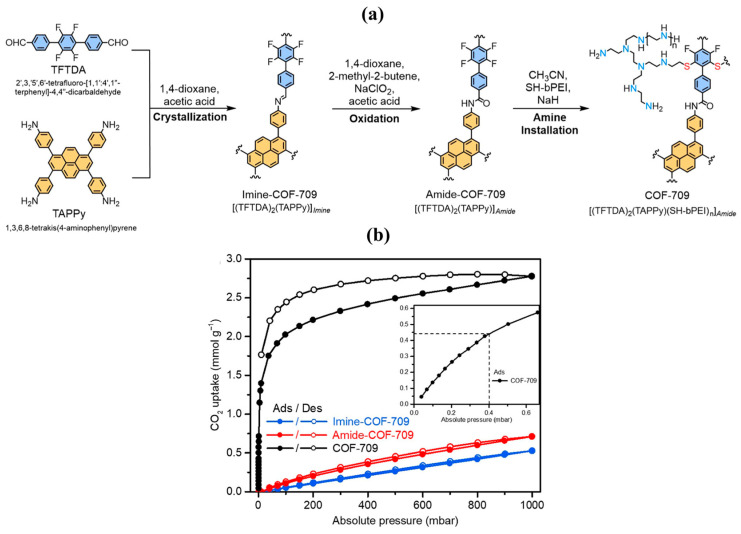
(**a**) Synthetic route for the preparation of COF-709 via imine condensation, linkage oxidation, and covalent PEI installation. (**b**) Single-component CO_2_ adsorption isotherms of imine-COF-709, amide-COF-709, and PEI-functionalized COF-709 at 25 °C. Reproduced from Ref. [64] with permission from ACS, 2024.

In another study [64], a polyethylenimine (PEI)-functionalized COF (COF-709) was developed for direct air capture of CO_2_ through a multistep post-synthetic modification strategy. As shown in Figure 12a, the material was first synthesized via Schiff-base condensation between 2′,3′,5′,6′-tetrafluoro-[1,1′:4′,1″-terphenyl]-4,4″-dicarbaldehyde and 1,3,6,8-tetrakis(4-aminophenyl)pyrene, forming a crystalline imine-linked COF (imine-COF-709) with an AA-stacked layered structure. PXRD analysis confirms its high crystallinity, with good agreement between experimental and simulated patterns, indicating well-ordered π–π stacking between layers. The pristine framework exhibits a high BET surface area of 1880 m^2^/g and a pore size of ~3.0 nm. Post-synthetic oxidation converts imine linkages into more stable amide bonds, which reduces the BET surface area to 1157 m^2^/g while maintaining crystallinity and improving chemical stability. In the next step, SH-functionalized branched PEI is covalently grafted into the pore channels via aromatic nucleophilic substitution, forming stable C–S linkages between the polymer and the COF backbone. This modification significantly increases the amine density (9.24 mmol/g), although it also leads to partial pore blocking and a reduction in accessible N_2_ porosity due to framework flexibility. Despite these changes in textural properties, the material shows excellent CO_2_ capture performance, reaching 0.44 mmol/g at 0.4 mbar (400 ppm CO_2_) and up to 1.77 mmol/g at higher pressures (Figure 12b). The isosteric heat of adsorption (Q_st_ = 41–50 kJ/mol) indicates strong chemisorption between CO_2_ molecules and amine sites. Dynamic breakthrough measurements under simulated air (400 ppm CO_2_) further show that CO_2_ uptake increases significantly with humidity, reaching 1.24 mmol/g at 75% RH compared with 0.48 mmol/g under dry conditions. This enhancement is attributed to water-assisted formation of bicarbonate species and faster amine–CO_2_ reaction kinetics. PXRD and cycling tests confirm excellent structural stability and full retention of adsorption capacity over multiple adsorption–desorption cycles at 95 °C regeneration temperature. Mechanistic studies using solid-state ^13^C NMR reveal that CO_2_ adsorption proceeds via carbamate formation under dry conditions and a mixed bicarbonate–carbamate pathway under humid conditions, which explains the humidity-dependent performance. Overall, this work demonstrates that covalent anchoring of polyamines within crystalline COFs through stable C–S linkages, combined with a hydrophobic framework environment, enables high-capacity, humidity-tolerant, and recyclable CO_2_ capture materials suitable for direct air capture applications [64].

The thiadiazole-functionalized COF (TH-COF-1) was synthesized via a bottom-up solvothermal Schiff-base condensation between 4,7-diaminobenzo[1,2,5]thiadiazole and 1,3,5-triformylbenzene, enabling the uniform incorporation of nitrogen-rich functional groups within the pore walls [65]. The framework adopts a 2D imine-linked layered structure with eclipsed AA stacking, as confirmed by PXRD, showing good crystallinity and close agreement between experimental and simulated patterns. Nitrogen sorption analysis shows a typical type-I isotherm, confirming its microporous nature, with a BET surface area of 684 m^2^/g and a total pore volume of 0.74 cm^3^/g. The material exhibits a pore size of ~1.1–1.5 nm, suggesting that thiadiazole incorporation effectively tunes pore confinement while still maintaining permanent porosity. At the same time, the presence of thiadiazole units introduces additional nitrogen sites directly into the pore environment. As a result, TH-COF-1 shows enhanced CO_2_ adsorption capacities of 128 mg/g (≈2.91 mmol/g) at 273 K and 97 mg/g (≈2.20 mmol/g) at 298 K (1 bar), clearly outperforming the non-functionalized analogue. This improvement can be linked to the combined effect of stronger dipole–quadrupole interactions between CO_2_ molecules and nitrogen sites, together with enhanced micropore confinement that favors adsorption at low pressure. The relatively high Q_st_ also supports stronger host–guest interactions, indicating that pore-wall functionalization plays a key role in tuning CO_2_ capture and separation behavior [65]. In general, functionalized COFs tend to shift the adsorption behavior from purely physisorption in pristine frameworks toward a more mixed physisorption–chemisorption regime. Introducing groups such as amines, heterocycles, or alkali metals can significantly improve CO_2_ affinity, although this often comes with a balance between stronger binding and regeneration energy requirements.

COF functionalization has proven to be an effective approach for enhancing CO_2_ capture by tailoring both the chemical environment and pore architecture. Each modification strategy contributes in a different way: amine-functionalized frameworks enable strong chemisorption through reversible carbamate or bicarbonate formation, alkali metal incorporation enhances framework polarity and strengthens electrostatic interactions, while heteroatom-rich structures improve dipole–quadrupole interactions and favor CO_2_ uptake at low pressures. In addition, polymer or alkyl chain modifications provide a flexible way to tune both pore size and surface chemistry, which directly affects adsorption behavior.

A key observation across these systems is the trade-off between introducing more active sites and maintaining accessible porosity. In many cases, increasing functional group density can lead to partial pore blocking or a decrease in surface area, which may reduce total adsorption capacity. Because of this, high-performing COFs depend on a careful balance between pore accessibility, functional group distribution, and structural stability. The final CO_2_ capture performance is therefore controlled by the combined effect of these factors, rather than any single structural feature.

#### 3.2.3. Composite COFs

Composite COFs have emerged as a promising class of materials for CO_2_ capture by integrating COFs with complementary porous or functional phases such as polymers, MOFs, or inorganic supports. This hybridization strategy effectively overcomes the intrinsic limitations of pristine COFs, including limited active sites and moderate CO_2_ affinity. By combining the high surface area and structural tunability of COFs with the functionality of guest components, composite systems enable synergistic enhancement of adsorption capacity, selectivity, and stability. For example, a metal-free COF–ionic polymer hybrid catalyst further demonstrates the role of confined ionic environments in CO_2_ utilization. In this system, imidazolium-based ionic liquids were polymerized inside triazine-based COF channels via a rapid 6 h ship-in-bottle strategy, forming PImBr@COF. The resulting material integrates multiple active sites, including Br^−^ nucleophiles, hydroxyl groups, imidazolium cations, and CO_2_-philic triazine units. This multifunctional structure enabled a CO_2_ adsorption capacity of 50.6 mg/g (≈1.15 mmol/g) at 298 K and 1 bar, along with an improved CO_2_/N_2_ selectivity of 24.7. More importantly, PImBr@COF exhibited outstanding catalytic performance in CO_2_ cycloaddition with epoxides, achieving a 99.0% yield of cyclic carbonate under metal-free, solvent-free conditions (100 °C, 2.0 MPa, 12 h). The catalyst maintained stable performance over 10 recycling cycles with negligible loss in activity. DFT calculations revealed that the confined ionic environment significantly lowers the activation barrier for epoxide ring opening from 57.69 kcal/mol (uncatalyzed) to 33.23 kcal/mol (catalyzed), confirming the key role of synergistic hydrogen bonding and nucleophilic activation [66].

Core–shell MOFs@COFs hybrids have shown significantly enhanced CO_2_ capture performance due to synergistic structural and chemical effects. In these systems, COFs are in situ grown on MOF cores through covalent linkage, forming stable interfacial structures that improve framework robustness and porosity (MOFs@COFs core–shell hybrid). For example, Zr-MOF@COF hybrids exhibit high BET surface areas up to 1301 m^2^/g and total pore volumes of 0.94 cm^3^/g, accompanied by heteroatom contents of 13.4% (N/S). These structural features generate additional interfacial micropores (1.48 nm), which enhance CO_2_ adsorption via micropore-filling effects. As a result, these hybrids demonstrate CO_2_ uptake up to 170 mg/g (≈3.86 mmol/g) at 273 K and 1 bar, significantly higher than the corresponding pristine MOFs and COFs. The enhanced performance is attributed to: (i) increased microporosity at the MOF/COF interface, (ii) strong electrostatic and acid–base interactions between CO_2_ and heteroatom-rich sites, and (iii) improved pore accessibility and diffusion pathways [67].

Carbon nanomaterials like CNTs and graphene serve as effective scaffolds to regulate COF crystal growth and enhance pore accessibility. Their integration improves gas diffusion pathways and CO_2_ uptake performance in composite systems. In a research study, Yoo and co-workers synthesized COF-5 on carbon nanotubes (CNTs) and graphene via a facile sonochemical one-pot reaction, forming CNT@COF-5 and graphene@COF-5 composite structures. The integration of COFs with carbon nanomaterials improved gas adsorption performance by enhancing pore architecture and dispersibility of the framework. In particular, CNT@COF-5 exhibited a higher CO_2_ uptake (1.42 wt%) compared to pristine COF-5 (1.25 wt%), despite the negligible mass contribution of CNTs, indicating a synergistic effect of nanoscale confinement and improved microcrystallite formation. The curved CNT surface promoted the growth of smaller COF domains, leading to increased pore volume and optimized microporosity, which enhanced CO_2_ accessibility [68].

**Figure 13:**
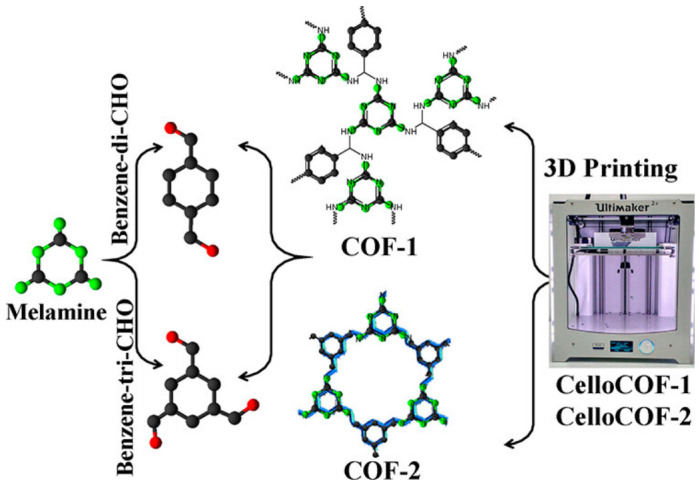
Schematic illustration of the synthesis of COF-1 and COF-2 followed by their fabrication into 3D-printed scaffold structures. Reproduced from Ref. [69] with permission from ACS, 2023.

Cellulose- and alginate-based polymers provide sustainable, functional scaffolds that enable the fabrication of structured COF-based CO_2_ adsorbents. As shown in Figure 13, a 3D-printed cellulose/alginate/COF composite (CelloCOF) was developed using direct ink writing (DIW) to overcome the aggregation and powder limitations of COFs. COF-1 and COF-2 were synthesized via Schiff-base polycondensation and incorporated into a TOCNF/SA (TOCNF: TEMPO (2,2,6,6-tetramethylpiperidine-1-oxyl)-assisted cellulose nanofiber; SA: sodium alginate) matrix, forming hierarchical porous scaffolds with macro–micropore architecture. BET analysis showed specific surface areas of 128 m^2^/g (ink COF-1), 39 m^2^/g (COF-2), 30 m^2^/g (3D CelloCOF-1), and 11 m^2^/g (3D CelloCOF-2), with dominant pore sizes at 3.1 and 20.4 nm. For CO_2_ adsorption, 3D CelloCOF-1 (freeze-dried) exhibited the highest capacity of 19.9 mg/g (≈0.45 mmol/g), compared to 8.5 mg/g (≈0.19 mmol/g) and 8.2 mg/g (≈0.19 mmol/g) for non-freeze-dried CelloCOF-1 and CelloCOF-2, respectively. The system showed good recyclability, with the adsorption capacity decreasing from 19.5 mg/g (≈0.44 mmol/g) to 16.2 mg/g (≈0.37 mmol/g) after repeated cycles, corresponding to a 16.9% loss. Adsorption followed a reversible physisorption mechanism with pressure-swing behavior and high CO_2_/N_2_ selectivity (491). The hierarchical structure prevented COF aggregation, improving accessibility of active sites and enhancing gas diffusion. Overall, 3D printing significantly improved COF processability while maintaining moderate CO_2_ capture performance and enabling scalable, sustainable adsorbent fabrication [69].

Chitosan-based bio-polymers facilitate strong integration with COFs, forming hierarchical aerogels with improved CO_2_ adsorption performance. A covalently integrated COF–polymer composite aerogel (COF-IL@chitosan) has been developed to enhance processability and CO_2_ capture performance of crystalline COFs by Ding et al. [70]. The material is constructed by crosslinking an ionic liquid-functionalized COF (COF-IL) with thiol-modified chitosan via a photoinduced thiol–ene reaction, followed by freeze-drying to generate a hierarchical macroporous aerogel. The composite retains the intrinsic crystallinity and porosity of COF-IL, while enabling high COF loading (up to 80 wt%) within a mechanically robust 3D architecture. Nitrogen sorption analysis shows a reduced but accessible BET surface area compared to pristine COF (≈103 m^2^/g vs. 291 m^2^/g), indicating partial pore blocking by the polymer matrix. The aerogel exhibits significant CO_2_ uptake (25.8 cm^3^/g, ≈ 1.05 mmol/g at 298 K) and high selectivity over N_2_ and CH_4_ due to imidazolium-based ionic liquid sites. The CO_2_ adsorption mechanism is governed by electrostatic interactions, quadrupole–dipole forces, and confinement effects within hierarchical pores [70].

From a broader perspective, composite COFs reflect a shift from single-component adsorption systems toward interfacial and hierarchical design strategies, where CO_2_ capture is controlled by multiple cooperative mechanisms rather than intrinsic porosity alone. In these systems, performance enhancement mainly originates from three key factors: (i) interfacial engineering between COFs and secondary phases, which introduces additional adsorption sites and modifies local polarity; (ii) hierarchical pore structures that facilitate diffusion and improve access to active sites; and (iii) functional synergy, where different components contribute complementary adsorption pathways such as physisorption, chemisorption, and electrostatic interactions. At the same time, introducing secondary phases can sometimes reduce intrinsic surface area, creating a trade-off between structural complexity and porosity retention.

Composite COFs generally show improved CO_2_ capture performance through the incorporation of polymers, MOFs, or ionic liquids within a single framework. Even when the BET surface area decreases partially, CO_2_ uptake and selectivity often improve due to interfacial adsorption effects, increased polarity, and the formation of confined active environments. The presence of hierarchical porosity also supports faster mass transfer and better accessibility of adsorption sites. In addition, heteroatom-rich domains and functional groups strengthen electrostatic and acid–base interactions with CO_2_ molecules. As a result, the performance of composite COFs depends on how well interfacial functionality is balanced with structural porosity, where hierarchical architecture and cooperative adsorption mechanisms together define the overall CO_2_ capture efficiency and practical potential.

#### 3.2.4. COF Membranes

From a processability point of view, COF membranes, both neat COF membranes as well as mixed-matrix membranes (MMMs), have gained widespread popularity in recent years. The gas permeability and separation selectivity of COF membranes tend to counter one another, and it is particularly challenging to integrate high permeability as well as selectivity in one COF membrane [71]. For instance, Liu et al. [72] developed multifunctional COF-based mixed-matrix membranes by incorporating polyethylene glycol (PEG)-modified hollow spherical COF fillers into a Pebax matrix to enhance CO_2_ separation performance. The pristine COF exhibited a high BET surface area of 1388.5 m^2^/g with a pore size of 3.2 nm, which decreased to 328.4–516.6 m^2^/g and 1.35–1.42 nm after PEG functionalization due to pore occupation by polymer chains. The hollow spherical morphology (50–500 nm diameter, 20–50 nm shell thickness) significantly reduced mass transfer resistance and provided additional gas transport pathways. Gas separation measurements revealed that Pebax–COF MMMs achieved a maximum CO_2_ permeability of 1044 Barrer and CO_2_/CH_4_ selectivity of 24 at 1 wt% loading. Upon PEG modification, the optimized Pebax-PEG200@COF membrane (3 wt%) exhibited an enhanced selectivity of 33.0 with a high CO_2_ permeability of 944 Barrer, surpassing the Robeson upper bound. Mechanistically, the performance improvement arises from the synergistic enhancement of diffusion selectivity (via pore size reduction) and solubility selectivity (via EO–CO_2_ interactions), with solubility selectivity increasing by ~29% compared to unmodified COF membranes. Additionally, the PEG chains improved interfacial compatibility with the Pebax matrix, increasing the optimal filler loading from 1 wt% to 3 wt% and suppressing non-selective defects. The membrane also demonstrated stable performance over 144 h, confirming its structural robustness for practical CO_2_ separation [72].

**Figure 14:**
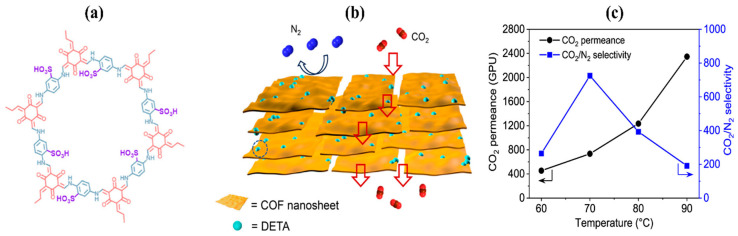
Schematic representation of the design of a COF membrane incorporating confined mobile carriers for efficient CO_2_ separation. (**a**) Chemical structure of TpPa-SO_3_H COF. (**b**) Schematic depiction of the COF membrane containing confined mobile CO_2_ carriers. (**c**) CO_2_/N_2_ separation performance of the TpPa-SO_3_H-DETA membrane at various temperatures. Reproduced from Ref. [73] with permission from ACS, 2023.

In a research study [73], as shown in Figure 14a–c, Wang et al. reported an ionic COF composite membrane with confined mobile amine carriers for efficient and stable CO_2_ separation under flue gas conditions. The TpPa-SO_3_H COF nanosheets, possessing negatively charged nanochannels (ζ of −48.5 mV) and micropores of 1.5 nm, were fabricated into ultrathin lamellar membranes with a thickness of ~190 nm via vacuum-assisted assembly. A typical mobile carrier, 2,5-diethylenetriamine (DETA), was introduced at an optimal concentration of 100 mg/mL, resulting in enhanced ionic conductivity (up to 11.39 μS/cm) and effective carrier confinement via strong electrostatic interactions. The optimized membrane exhibited a CO_2_ permeance of 2347 GPU and a CO_2_/N_2_ selectivity of 191 at 90 °C under humid flue gas conditions (10/90 vol%, RH >99%). At a lower temperature (70 °C), a maximum selectivity of 725 with a permeance of 735 GPU was achieved, highlighting the trade-off between diffusion and reaction kinetics. Mechanistically, CO_2_ transport is governed by a facilitated transport pathway involving reversible carbamate formation with amine carriers, coupled with diffusion through 2D nanochannels. Importantly, the membrane maintained stable performance over 310 h with <5% fluctuation, attributed to strong electrostatic confinement (binding energy ≈ 170.66 kJ/mol) that effectively suppresses carrier leaching [73].

In another study [74], Ying et al. fabricated ultrathin and oriented COF-LZU1 films with controllable thicknesses ranging from 15 to 200 nm, where an optimized thickness of approximately 55 nm provided an ideal balance between mechanical integrity and mass transport resistance. The intrinsic porosity and ordered nanochannels (~1.8 nm pore size) of the COF layer significantly reduced gas transport resistance, contributing only ~7.37% to the overall membrane resistance. As a result, the optimized XLPEG/COF-LZU1/PAN membrane exhibited a high CO_2_ permeance of 1843 GPU and a CO_2_/N_2_ selectivity of 28.2, surpassing the industrial benchmark values (>1000 GPU and >20, respectively). Notably, the COF-based gutter layer demonstrated superior permeability (~25,000 GPU) compared to conventional materials such as PDMS, while also mitigating physical aging effects over at least one month. Furthermore, under humid conditions (85% RH) and elevated temperature (333 K), the membrane showed enhanced CO_2_ permeance (+11%) and selectivity (+12%), indicating favorable interaction with water vapor. These findings highlight the critical role of COF-based gutter layers in enabling ultrathin selective layers (~120 nm) and achieving high-performance, stable CO_2_ capture membranes, making them promising candidates for next-generation composite COF membrane design [74].

The use of azine-linked COF (ACOF-1) as fillers in MMMs has demonstrated a strong dependence of separation performance on the nature of the polymeric matrix. In this study, MMMs were fabricated using three polymers with distinct transport properties, namely Matrimid^®^, Polyactive™, and 6FDA:DAM, with COF loadings up to 16 wt%. ACOF-1 exhibited a high surface area (~1310 m^2^/g) and significant CO_2_ adsorption capacity (up to 4.6 mmol/g at 298 K and 5 bar), along with CO_2_/N_2_ adsorption selectivity of ~13–15. For Matrimid^®^-based MMMs, the incorporation of 16 wt% COF resulted in a notable increase in CO_2_ permeability from 9.5 to 17.7 Barrer and an improvement in CO_2_/N_2_ selectivity from 29 to 35, indicating synergistic enhancement due to good polymer–filler compatibility. In contrast, Polyactive™-based MMMs showed decreased permeability due to partial pore blockage by flexible polymer chains, while 6FDA:DAM-based membranes exhibited slight selectivity improvement but limited permeability gains due to the already high intrinsic permeability of the polymer [75].

In 2021, Jiang and co-workers developed [76] an ion-exchange strategy to achieve a uniform distribution of facilitated transport species within cationic COF-based membranes. Initially, TpTGCl nanosheets were prepared from 1,3,5-Triformylphloroglucinol (T_p_) and triaminoguanidinium chloride (TG_Cl_) precursors, after which a two-step ion-exchange process was used to substitute chloride ions with borate, yielding TpTG_B_ nanosheets with active transport functionality. To construct defect-free ultrathin membranes, small amounts of GO nanosheets were incorporated and assembled onto a porous polyacrylonitrile support via vacuum filtration. The GO sheets interacted strongly with TpTG_B_ through electrostatic attraction and hydrogen bonding, which helped eliminate interfacial voids between COF layers and significantly improved membrane integrity. The resulting selective layer thickness ranged from approximately 20 nm to 60 nm, depending on GO content. Gas separation performance was evaluated using a CO_2_/CH_4_ (30/70 vol%) mixture under humidified conditions. The pristine TpTG_B_ membrane showed a high CO_2_ permeance of 210 GPU but a relatively low CO_2_/CH_4_ selectivity of 11, indicating the presence of structural defects. After introducing GO, selectivity improved substantially. The optimized TpTG_B_–GO membrane with a TpTG_B_:GO ratio of 15:1 achieved a CO_2_ permeance of 164.2 GPU and a CO_2_/CH_4_ selectivity of 27. Control experiments using a chloride-based COF (TpTG_Cl_) confirmed the role of borate carriers. The TpTG_Cl_ membrane exhibited extremely high permeance (>10,000 GPU) but almost no selectivity (~1), indicating non-selective transport. When GO was added, defects were partially blocked, improving selectivity while reducing permeance. Overall, the performance enhancement resulted from two synergistic effects: (i) GO nanosheets repairing structural defects and regulating transport pathways, and (ii) borate ions acting as facilitated transport carriers that preferentially accelerate CO_2_ diffusion through the membrane channels [76].

In COF membranes, CO_2_ separation is controlled by a balance between solution–diffusion, facilitated transport, and molecular sieving mechanisms, where nanochannel design and interfacial compatibility are crucial for addressing the permeability–selectivity trade-off. The contribution of each transport pathway strongly depends on the degree of crystallinity and the continuity of the COF nanochannels. In general, higher crystallinity helps reduce non-selective voids and supports more size-selective transport, while structural defects can disturb molecular sieving and may lead to reduced selectivity or non-selective leakage.

COF-based membranes, including both neat COFs and mixed-matrix systems, provide a highly tunable platform for CO_2_ separation, with competitive permeability and selectivity arising from their ordered channels, CO_2_-philic functional groups, and engineered interfaces. This performance is mainly driven by three synergistic factors: (i) well-defined crystalline channels that enable selective diffusion, (ii) chemical functionalities that regulate CO_2_ solubility through dipole–quadrupole and acid–base interactions, and (iii) interfacial engineering that helps minimize non-selective defects and maintain transport continuity. At the same time, these advantages are closely linked to an inherent trade-off between permeability and selectivity, which often becomes more evident when functional complexity or filler content increases, sometimes at the cost of reduced crystallinity or blocked transport pathways. Another important point is long-term stability under humid conditions, since water molecules can compete for adsorption sites and alter transport behavior, especially in amine- or ionically functionalized systems.

Overall, CO_2_ transport and adsorption in COF membranes are governed by a combined effect of pore structure, crystallinity, and chemical functionality. Ultramicroporous COFs (<1 nm) generally enhance CO_2_ affinity through confinement effects, which strengthen van der Waals and electrostatic interactions at low pressure. On the other hand, larger-pore systems tend to improve mass transport but usually show weaker interaction strength and lower selectivity. The incorporation of heteroatoms such as N, O, and F further increases framework polarity and strengthens CO_2_ interactions. Functionalization can shift the behavior from weak physisorption toward stronger interactions, particularly in amine-based systems. However, excessive functionalization or pore blocking can reduce accessible porosity and disturb structural order, affecting both diffusion and adsorption. Therefore, the design of COF membranes requires a careful balance between nanochannel crystallinity, interfacial compatibility, and transport pathways, where separation performance ultimately depends on the interplay between diffusion, structural order, and CO_2_-specific interactions.

#### 3.2.5. Comparative Summary and Structure–Property Relationship

The comparative analysis in Table 1 shows a clear relationship between structure, properties, and CO_2_ capture performance in COFs. In pristine COFs, CO_2_ uptake is mainly governed by physisorption, where pore size distribution and micropore confinement play the most important roles. For example, JUC-610 and 3D-CageCOF-1 both show that well-defined microporosity together with high surface area can significantly improve CO_2_ adsorption, especially at low pressure.

In functionalized COFs, the performance is generally higher due to the presence of polar or reactive groups such as amines, ionic functionalities, and heteroatom-rich frameworks. These groups strengthen the interaction between CO_2_ molecules and the framework and usually lead to higher Q_st_. In some cases, the adsorption behavior also shifts from simple physisorption toward stronger or more cooperative interactions. For instance, amine-functionalized materials like TPA-4C-NH_2_ clearly show how increasing pore polarity and functional group density can improve CO_2_ uptake.

The relationship between BET surface area and CO_2_ uptake is not straightforward. Although higher surface area and preserved porosity generally help gas diffusion and accessibility, post-synthetic modifications often reduce BET values. Interestingly, this reduction does not always lead to lower performance. In many cases, the introduction of more active adsorption sites can compensate for the loss in surface area. However, if pore blockage or structural disorder occurs, the overall uptake may decrease. This indicates that chemical environment and pore accessibility are often more important than surface area alone.

Composite COFs further highlight the role of structural engineering. By combining COFs with polymers, ionic liquids, or MOF-derived components, it is possible to improve diffusion pathways and create additional adsorption sites. As a result, both CO_2_ uptake and selectivity can be enhanced through synergistic effects.

In membrane systems, the mechanism is different. Instead of equilibrium adsorption, CO_2_ separation depends more on transport through the membrane. Here, factors such as channel size, orientation of the framework, and film thickness become the key parameters controlling permeance and selectivity, as seen in high-performance systems like TpPa-SO_3_H-DETA.

Overall, the results suggest that CO_2_ capture in COFs cannot be explained by a single factor. It is the combined effect of pore structure, surface chemistry, and framework stability that determines the final performance. A balanced design between these parameters is therefore essential for achieving efficient CO_2_ adsorption and separation.

## 4. Structure–Performance Correlations in COFs for CO_2_ Capture

**Figure 15:**
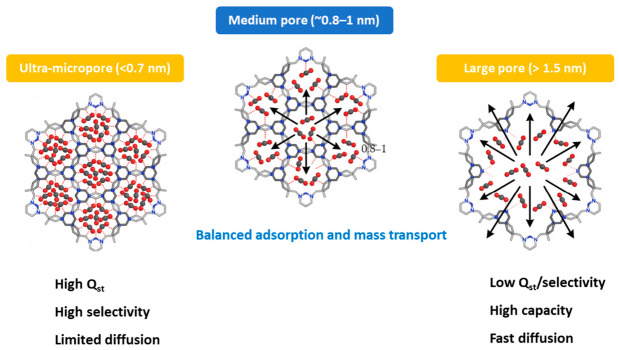
Pore size-dependent CO_2_ adsorption behavior in COFs, illustrating the trade-off between adsorption strength (Q_st_), selectivity, and diffusion kinetics.

A clear relationship can be seen between pore size, Qst, and CO_2_/N_2_ selectivity in COFs. Ultramicroporous COFs (pore size below ~0.7 nm) usually show higher selectivity because the confined space strengthens CO_2_–framework interactions. In these small pores, adsorption potentials overlap more strongly, which enhances electrostatic and quadrupole-driven interactions with CO_2_ and leads to higher Q_st_ values. This is mainly a thermodynamic effect, since CO_2_ is more strongly stabilized inside the pores. However, this strong confinement also slows down diffusion, creating kinetic limitations that can reduce mass transport and limit usable capacity in practical conditions. On the other hand, COFs with larger pores (>1.5 nm) allow easier diffusion and better accessibility of the pore volume, which supports faster mass transport and higher uptake at elevated pressures (Figure 15). But in this case, the weaker confinement reduces interaction strength, resulting in lower Q_st_ values and generally reduced selectivity. This shows that CO_2_ capture is not controlled by pore size alone, but rather by a balance between adsorption strength (thermodynamics) and diffusion efficiency (kinetics). This trade-off can be partly adjusted through chemical functionalization. Polar groups such as –NH_2_ and –OH introduce localized electrostatic interactions and hydrogen-bonding sites that strengthen CO_2_ affinity, even in larger-pore systems. In this way, chemical functionality helps compensate for weaker confinement and allows better control over both selectivity and transport behavior. From an application point of view, ultramicroporous COFs are more suitable for low-pressure or dilute CO_2_ capture, where strong adsorption is essential. In contrast, larger-pore COFs are better suited for high-pressure or high-flux conditions, where fast diffusion becomes more important. Overall, effective CO_2_ capture requires a balanced design of pore structure and surface chemistry, tailored to the specific operating conditions rather than relying on a single structural parameter.

## 5. Comparison with Other Adsorbents

As shown in Table 2, COFs exhibit exceptional structural tunability and, in many cases, high CO_2_ selectivity compared with other porous adsorbents. However, zeolites, activated carbons, and amine-functionalized silicas remain competitive because of their lower cost, greater process maturity, and, in some cases, stronger low-pressure capture performance. Overall, COFs occupy an intermediate position between mature sorbents and highly tunable but often more expensive frameworks such as MOFs.

## 6. Practical Considerations for COF-Based CO_2_ Adsorption

While COFs have shown strong CO_2_ adsorption performance under ideal laboratory conditions (pure CO_2_, dry gas, and low temperature), their real-world application depends on how they perform under more practical operating environments. Several key factors become important in this context, including moisture resistance, mixed-gas selectivity, cyclic stability, and dynamic breakthrough behavior. In particular, moisture is one of the main challenges in real flue gas systems. Water vapor can compete with CO_2_ for adsorption sites and, in some cases, even weaken or damage the framework, especially in boron-linked COFs. On the other hand, more stable linkages such as β-ketoenamine and triazine-based structures generally show better resistance to hydrolysis and can maintain their porosity under humid conditions. Still, comprehensive studies across different humidity levels are not yet fully established. In real industrial gas streams, CO_2_ is always mixed with other gases such as N_2_, O_2_, and water vapor, so CO_2_/N_2_ selectivity becomes a critical parameter. In many cases, mixed-gas experiments and IAST calculations show lower selectivity compared to single-component measurements, which highlights the importance of evaluating competitive adsorption rather than relying only on pure-gas data. Another important aspect is cyclic stability. Although many COFs maintain their structure over a few adsorption–desorption cycles, long-term performance can still be affected by gradual framework degradation or loss of functional activity under repeated regeneration conditions. Extended cycling data under realistic operating conditions are still relatively limited. Finally, breakthrough experiments provide a more realistic picture of separation performance because they simulate continuous flow and include mass transfer effects. However, such studies are still not widely reported for COF systems, especially under humid and multicomponent gas conditions, which are essential for understanding scale-up potential. Therefore, addressing these practical challenges is essential to close the gap between laboratory performance and real industrial CO_2_ capture applications [93,94,95,96].

## 7. Conclusions

The main conclusions drawn from this review are summarized as follows:

- COFs have developed into a highly versatile class of porous materials for CO_2_ capture and separation owing to their tunable pore structures, low density, and flexible chemical design.
- Across pristine, functionalized, composite, and membrane-based systems, CO_2_ adsorption is governed by a combination of pore confinement, surface chemistry, and framework architecture.
- In pristine COFs, adsorption performance is mainly controlled by micropore size, pore volume, and accessible surface area, where CO_2_ uptake is dominated by physisorption within ordered porous channels.
- Functionalized COFs introduce additional interaction sites, including amine groups, heteroatoms, ionic functionalities, and metal-based centers, which enhance CO_2_ affinity and often result in mixed physisorption–chemisorption behavior.
- Composite COFs and membrane-based architectures further expand the applicability of COFs by improving mass transport, interfacial interactions, structural robustness, and processability.
- CO_2_ adsorption in COFs cannot be explained by a single structural parameter. Instead, adsorption performance results from the combined influence of pore structure, chemical functionality, crystallinity, framework polarity, and stability.
- Increasing functional group density often enhances adsorption affinity but may simultaneously reduce accessible porosity and diffusion rates.
- Similarly, ultramicropores improve CO_2_ binding strength and selectivity, whereas larger pores facilitate molecular transport but may weaken adsorption interactions.
- Therefore, the design of high-performance COFs requires a careful balance between adsorption capacity, selectivity, diffusion kinetics, and regeneration efficiency rather than simply maximizing surface area or functional group density.

## 8. Future Perspectives

The key future research directions for advancing COF-based CO_2_ capture technologies are summarized below:

- The development of humidity-resistant COFs remains one of the most important research priorities because water vapor is inevitably present in practical gas streams and can significantly influence adsorption behavior.
- Improving hydrolytic stability through robust linkage chemistries, such as β-ketoenamine, triazine, and imide linkages, together with hydrophobic pore engineering, is expected to enhance long-term operational performance.
- Direct air capture (DAC) represents a particularly promising but challenging application area. Future DAC-oriented COFs should combine strong CO_2_-binding sites, ultramicroporous confinement, and low regeneration energy requirements.
- Scalable synthesis and processing remain major obstacles to commercialization. Future efforts should focus on developing cost-effective synthetic routes and producing industrially relevant forms such as pellets, monoliths, membranes, and structured adsorbents.
- Maintaining crystallinity, porosity, and adsorption performance during large-scale processing will be critical for practical deployment.
- Computational screening, molecular simulations, and machine learning techniques are expected to accelerate the discovery of next-generation COFs by enabling rapid prediction of adsorption behavior and guiding experimental design.
- Advanced COF-based membranes offer considerable potential for energy-efficient CO_2_ separation; however, challenges related to interfacial compatibility, non-selective void formation, and defect control must still be addressed.
- The development of facilitated transport mechanisms, engineered nanochannels, and ultrathin selective layers may help overcome the permeability–selectivity trade-off.
- Ultimately, future progress in COF-based CO_2_ capture will require an integrated approach combining molecular design, stability engineering, scalable manufacturing, computational guidance, and process-level evaluation to bridge the gap between laboratory research and industrial implementation.

## Figures and Tables

**Table 1:** Expanded comparison of representative COFs for CO_2_ capture and separation.

Category	COF System	BET Surface Area (m^2^/g)	Pore Size (nm)	CO_2_ Uptake and CO_2_ Permeability	Selectivity	Q_st_ (kJ/mol)	Main Adsorption Mechanism/Key Feature	Ref.
**Pristine**	JUC-610	2072	1.5	2.50 mmol/g (273 K); 1.46 mmol/g (298 K)	—	21.9	Micropore confinement and high surface area	[55]
**Pristine**	COF-H1	510	1.31	1.29 mmol/g (273 K)	—	32.38	Pyrazine N sites and hydroxyl groups	[56]
**Pristine**	COF-H2	625	1.18	1.50 mmol/g (273 K)	—	26.83	Nitrogen-rich pore environment	[56]
**Pristine**	CIN	722	Microporous	3.32 mmol/g (273 K); 2.50 mmol/g (298 K)	211 (273 K)	42.4	Triazine and piperazine N sites	[57]
**Pristine**	TP-TFPB-COF	871	1.42	1.96 mmol/g (298 K)	2.04	23.4	Fluorinated pore environment	[58]
**Pristine**	TP-NFPB-COF	1089	1.48	1.62 mmol/g (298 K)	2.86	12.9	Highly fluorinated pores	[58]
**Pristine**	3D-CageCOF-1	1040	0.56–0.88	4.46 mmol/g (273 K); 2.43 mmol/g (298 K)	—	—	Cage-based flexible microporous framework	[59]
**Functionalized**	TPA-4C-NH_2_	~1800	~1.0	5.26 mmol/g (273 K); 3.41 mmol/g (298 K)	+121% vs. pristine	Increased	Amine-rich confined pores	[61]
**Functionalized**	TpPa-COOLi	96	1.37	2.65 mmol/g (273 K)	—	—	Li^+^ induced polarization	[62]
**Functionalized**	TpPa-COONa	101	1.26	2.40 mmol/g (273 K)	—	—	Electrostatic interaction	[62]
**Functionalized**	TpPa-COOK	65	1.18	2.42 mmol/g (273 K)	—	—	Alkali metal functionalization	[62]
**Functionalized**	TpPa-SO_3_Li	65	1.35	2.13 mmol/g (273 K)	—	—	Polar sulfonate sites	[62]
**Functionalized**	TpPa-SO_3_Na	67	1.34	2.08 mmol/g (273 K)	—	—	Framework polarization	[62]
**Functionalized**	TpPa-SO_3_K	65	1.27	2.05 mmol/g (273 K)	—	—	Ionic adsorption sites	[62]
**Functionalized**	TaTp-COF	114	1.15	5.0 mmol/g	—	—	Secondary amine chemisorption	[63]
**Functionalized**	PEI@COF-709	1157	~3.0	1.77 mmol/g	—	41–50	Carbamate/bicarbonate formation	[64]
**Functionalized**	TH-COF-1	684	1.1–1.5	2.91 mmol/g (273 K); 2.20 mmol/g (298 K)	—	—	Thiadiazole N-rich adsorption sites	[65]
**Composite**	PImBr@COF	—	Hierarchical	1.15 mmol/g	24.7	—	Ionic polymer confinement	[66]
**Composite**	Zr-MOF@COF	1301	1.48	3.86 mmol/g	—	—	Interfacial micropores and heteroatoms	[67]
**Composite**	CNT@COF-5	—	Micro/mesoporous	1.42 wt%	—	—	Improved diffusion pathways	[68]
**Composite**	Graphene@COF-5	—	Micro/mesoporous	Improved vs. pristine	—	—	Enhanced pore accessibility	[68]
**Composite**	3D CelloCOF-1	30	3.1, 20.4	0.45 mmol/g	491	—	3D-printed hierarchical scaffold	[69]
**Composite**	3D CelloCOF-2	11	Hierarchical	0.19 mmol/g	—	—	Bio-based porous architecture	[69]
**Composite**	COF-IL@Chitosan aerogel	103	Hierarchical	1.05 mmol/g (298 K)	High vs. N_2_/CH_4_	—	Ionic liquid-functionalized aerogel	[70]
**Membrane**	Pebax/COF MMM	—	3.2	1044 Barrer	24 (CO_2_/CH_4_)	—	Hollow COF fillers	[72]
**Membrane**	Pebax/PEG200@COF MMM	—	1.35–1.42	944 Barrer	33 (CO_2_/CH_4_)	—	PEG-assisted selective transport	[72]
**Membrane**	TpPa-SO_3_H-DETA	~190 nm film	1.5 nm channels	2347 GPU	191 (90 °C); 725 (70 °C)	—	Facilitated transport membrane	[73]
**Membrane**	COF-LZU1/PAN	—	1.8	1843 GPU	28.2	—	Ultrathin oriented COF layer	[74]
**Membrane**	ACOF-1/Matrimid MMM	1310	Microporous	17.7 Barrer	35	—	Polymer–COF synergy	[75]
**Membrane**	TpTG_B_–GO	20–60 nm film	Layered channels	164.2 GPU	27 (CO_2_/CH_4_)	—	Borate facilitated transport + GO defect healing	[76]

**Table 2:** Comparative performance of COFs and representative porous adsorbents for CO_2_ capture.

Adsorbent	CO_2_ Uptake (0.15 bar, 298 K)	CO_2_ Uptake (1 bar, 298 K/273 K)	Q_st_	Stability (Humidity/Cycles)	Strengths	Limitations	References
COFs	~0.5–2 mmol/g for typical COFs; higher for amine-functionalized or ultramicroporous COFs	~2–6 mmol/g at 298 K and ~4–10 mmol/g at 273 K, depending on framework chemistry and pore size	~20–50 kJ/mol; functionalized COFs usually fall in this range	Moderate to high; better performance in COFs with stable linkages and proper functionalization	Tunable pores, functionalization, low density	Moderate to high synthesis cost; scale-up still developing	[77]
MOFs	~1–4 mmol/g at 0.15 bar for CO_2_-suitable MOFs	~4–10 mmol/g at 298 K and in some cases up to ~12 mmol/g at 273 K	~25–60 kJ/mol depending on open metal sites and functional groups	Often poor under humid conditions; many MOFs are moisture-sensitive	Highest capacity, ultra-high surface area	Expensive, moisture sensitivity	[78,79,80]
Zeolites (e.g., 13X)	~2–4 mmol/g at 0.15 bar for common CO_2_-capture zeolites	~3–7 mmol/g under dry conditions; usually higher at 273 K	~30–50 kJ/mol	Poor in the presence of moisture; strong water competition	Cheap, industrially mature, strong selectivity	Water competition, capacity loss in humid gas	[81]
Activated carbon	Typically <1 mmol/g at 0.15 bar for generic materials; higher values are possible in optimized carbons	~2–5 mmol/g at 298 K; higher at 273 K	~10–30 kJ/mol	Excellent moisture stability and good cycling robustness	Low cost, scalable, hydrophobic	Poor selectivity, weak low-pressure uptake	[78,82,83]
Amine-functionalized solids	~2–5 mmol/g at low pressure for materials with high amine loading	~3–6 mmol/g depending on amine type and support	~60–100 kJ/mol; chemisorption dominates	Good, but oxidative degradation and amine loss may occur	High selectivity, works well at low pressure	High regeneration energy	[84,85]
Mesoporous silica (amine-grafted)	~1–3 mmol/g at 0.15 bar	~2–4 mmol/g at 1 bar/273 K	~50–90 kJ/mol	Moderate; risk of amine leaching exists	Tunable surface chemistry	Limited capacity compared with MOFs/COFs	[85]
POPs	~0.5–2 mmol/g	~2–5 mmol/g	~20–40 kJ/mol	Good	High stability, easy synthesis	Lower capacity than MOFs	[86]
Carbon nanotubes/graphene	Typically <1 mmol/g for undoped materials	~2–6 mmol/g for porous or doped samples	~10–30 kJ/mol	Excellent	Conductivity, stability	Low selectivity unless doped	[87,88,89]
Metal oxides (e.g., CaO, MgO)	2–10 mmol/g in high-temperature systems.	More relevant for high-T capture than for room-temperature comparison	>100 kJ/mol	Poor cyclic stability due to sintering	Suitable for high-temperature capture	High regeneration energy	[90]
Ionic liquids (supported)	~1–3 mmol/g	~2–4 mmol/g	~40–80 kJ/mol	Good	Tunable chemistry	High cost, viscosity issues	[91]
Molecular sieves/CMS	~1–3 mmol/g	~2–5 mmol/g	~20–40 kJ/mol	Good	Fast kinetics	Limited tunability	[92]

* Values are representative ranges compiled from the literature and may vary with framework chemistry, pore structure, operating pressure, temperature, and humidity.

## Data Availability

No new data were created in this study. Data sharing is not applicable to this article.
